# Identification of Novel Regulatory Genes in APAP Induced Hepatocyte Toxicity by a Genome-Wide CRISPR-Cas9 Screen

**DOI:** 10.1038/s41598-018-37940-6

**Published:** 2019-02-04

**Authors:** Katherine Shortt, Daniel P. Heruth, NiNi Zhang, Weibin Wu, Shipra Singh, Ding-You Li, Li Qin Zhang, Gerald J. Wyckoff, Lei S. Qi, Craig A. Friesen, Shui Qing Ye

**Affiliations:** 1 Division of Experimental and Translational Genetics, University of Missouri Kansas City School of Medicine, Kansas City, USA; 2 Division of Gastroenterology, Hepatology, Nutrition, Children’s Mercy Kansas City, Kansas City, MO USA; 3 Department of Biomedical and Health Informatics, University of Missouri Kansas City School of Medicine, Kansas City, MO USA; 4 Department of Biomedical Sciences, University of Missouri Kansas City School of Medicine, Kansas City, MO USA; 5 Division of Cell Biology and Biophysics, University of Missouri Kansas City School of Biological Sciences, Kansas City, MO USA; 6 Division of Molecular Biology & Biochemistry, University of Missouri Kansas City School of Biological Sciences, Kansas City, MO USA; 7 Department of Pediatrics, Tangdu Hospital, The Fourth Military Medical University, Xian, China; 8 Department of Bioengineering, Department of Chemical and Systems Biology, ChEM-H, Stanford University, Stanford, CA 94305 USA; 9 Present Address: Precision Genomics, Intermountain Healthcare, St. George, UT 84790 USA

## Abstract

Acetaminophen (APAP) is a commonly used analgesic responsible for more than half of acute liver failure cases. Identification of previously unknown genetic risk factors would provide mechanistic insights and novel therapeutic targets for APAP-induced liver injury. This study used a genome-wide CRISPR-Cas9 screen to evaluate genes that are protective against, or cause susceptibility to, APAP-induced liver injury. HuH7 human hepatocellular carcinoma cells containing CRISPR-Cas9 gene knockouts were treated with 15 mM APAP for 30 minutes to 4 days. A gene expression profile was developed based on the 1) top screening hits, 2) overlap of expression data from APAP overdose studies, and 3) predicted affected biological pathways. We further demonstrated the implementation of intermediate time points for the identification of early and late response genes. This study illustrated the power of a genome-wide CRISPR-Cas9 screen to systematically identify novel genes involved in APAP-induced hepatotoxicity and to provide potential targets to develop novel therapeutic modalities.

## Introduction

APAP is a widely used medication and is responsible for ~50% of acute liver failure (ALF) cases in the US and Great Britain^1,2^. It is the top risk factor for acute liver injury (ALI) and ALF in the US and Great Britain and in the top 3 in China^3^. The recommended maximum daily dose of APAP is 4 g for adults, with a single dose of just 7.5–10 g causing acute toxicity^4^. Ultimately, 36% cases of APAP induced ALF survive if no liver transplant occurs and patients who receive a liver transplant have a 75% survival rate.10% of APAP is processed in the liver by cytochrome-P450 to produce a toxic metabolite N-acetyl-p-benzo-quinone imine (NAPQI). Glutathione is used to convert NAPQI to a non-toxic substrate. When NAPQI levels are high, glutathione is depleted, causing an immune response and necrosis, which characterize acute liver failure. Current treatments of APAP-induced ALF focus on clearing excess APAP and replenishing glutathione and are only effective during a very short window of time post-overdose. The etiology of APAP-induced ALF is complex and not fully understood, particularly for cases that present more than 8 hours post-ingestion^5^. These cases are extremely troublesome because the liver injury can be asymptomatic for 24–48 hours. When the canonical APAP clearance pathways including metabolism via CYP2E1 are overwhelmed or low-functioning, redundant or accessory pathways may help to preserve function^6^. Furthermore, there is evidence that APAP overdose may cause cell death by multiple mechanisms^7^. Genetic predisposition may play a significant role in an individual’s susceptibility to APAP induced hepatotoxicity^8^. There is a demonstrated need for improved modalities of risk assessment, diagnosis, and therapeutics.

Microarray and “omics” approaches have widely been used to identify genes acting in APAP-induced injury^8–13^. These studies measure the changes in gene expression post-drug treatment using RNA sequencing or gene expression profiling, however the genes identified may not be causal. Previous screens of various diseases were accomplished using gene knockdown by RNA interference (RNAi), resulting in incomplete gene knockout and limiting the applications of the method^14–16^. Zinc finger nucleases (ZFNs) and transcription activator-like effector nucleases (TALENs) produce double-stranded breaks, however it is difficult to target multiple targets simultaneously with these methods^17–21^. CRISPR-Cas9 pooled lentiviral libraries provide stable, genome-wide gene knockout alternative that makes possible direct assessment of gene function that previous methods have not achieved^21,22^. In addition to the CRISPR-Cas9 pooled gene knockout libraries, genome-wide CRISPR/Cas9 SAM (Synergistic Activation Mediator) and CRISPRi (CRISPR interference) sgRNA libraries enable robust, multi-approach CRISPR screens^23–28^. Similarly to RNAi screens, in a CRISPR-Cas9 knockout library a positive screen identifies enriched gene knockouts after drug treatment. These genes potentially increase susceptibility to the treatment condition. A negative screen identifies depleted gene knockouts after drug treatment. These genes are potentially essential to survival of the treatment condition. The genome-wide CRISPR-Cas9 knockout screen has successfully identified genes contributing to a large variety of mechanisms, including essential genes and genes that conferred loss of resistance to vemurafenib in a melanoma model^23,29^.

This study builds on the existing CRISPR-Cas9 screening technology and applies it to a novel study of APAP-induced hepatotoxicity. We performed a genome-scale CRISPR-Cas9 screen of APAP toxicity (30 minutes–4 days) using the GeCKOv2 sgRNA library. We identified groups of genes and biological pathways that are protective against APAP, and other genes that increase susceptibility to injury. An understanding of which genes act in protecting from or enhancing injury at different times can better inform candidate gene discovery and elucidate the molecular pathways acting in response to APAP. By cross-referencing these data with existing gene expression data on APAP overdose in Humans and mice, we validated findings from our screen and connected the effect of CRISPR-Cas9 gene knockout on drug metabolism with the effect of drug on gene expression. From these data, we hypothesized the role of novel genes and validate the functional effect of knockdown of select candidate genes. These findings inform changes in the diagnostic and therapeutic modalities employed at the patient, with the ultimate goal of improving outcomes of APAP-induced ALF.

## Results

### Development of screening strategy and preparation of cell lines

**Figure 1:**
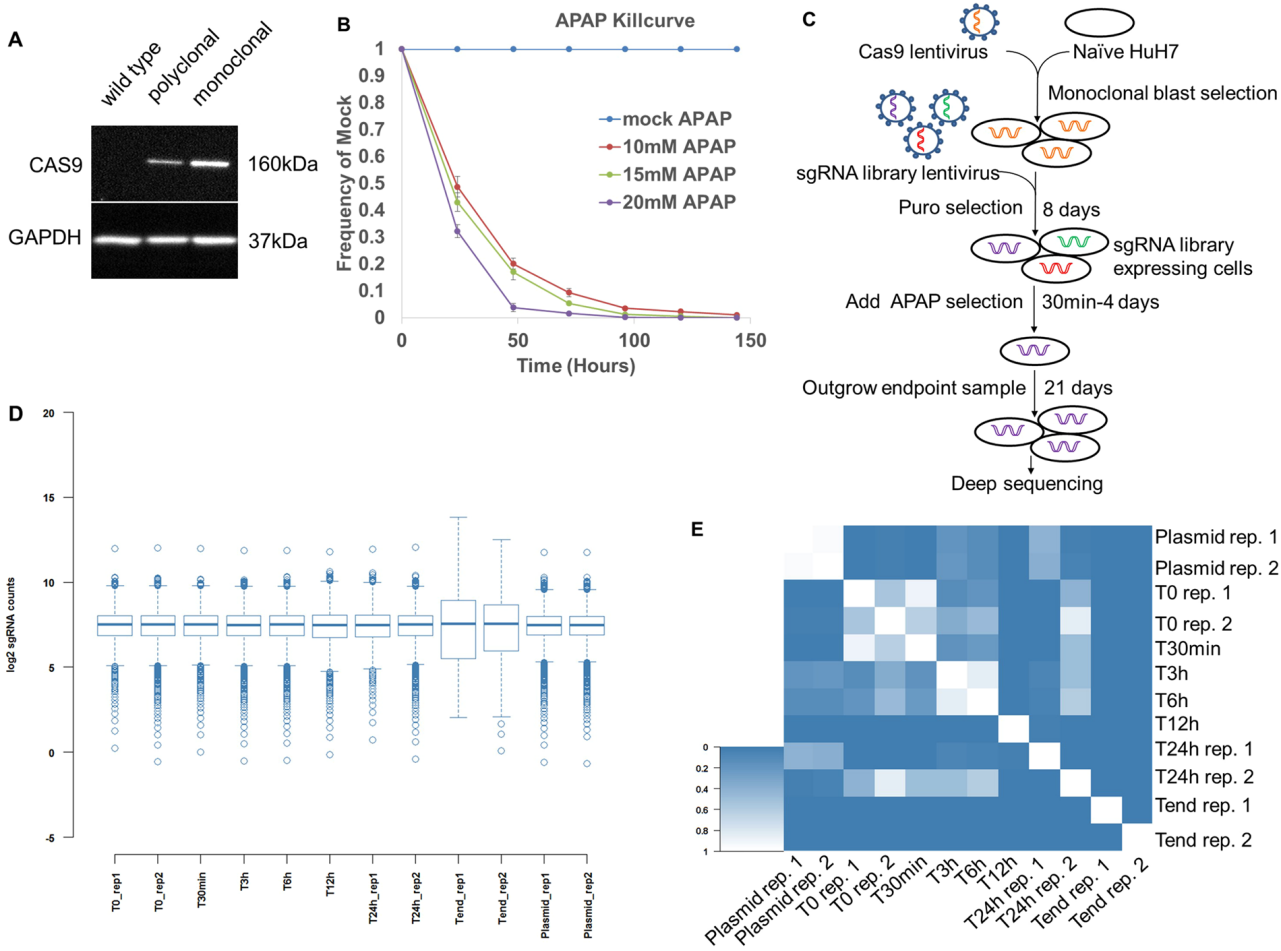
Genome-scale positive and negative screening using CRISPR/Cas9. (**A**) Expression levels of Cas9 in polyclonal and Monoclonal HuH7-Cas9 cell line. Full-length western blots are presented in Supplementary Fig. 1. (**B**) Relative growth of HuH7-Cas9/GuidePuro when treated with and without APAP. (**C**) Timeline of APAP resistance screen in HuH7 hepatocellular carcinoma cells. (**D**) Box-plot showing the distribution of log_2_ median-normalized sgRNA read count frequencies of the plasmid library (plasmid) and post-lentiviral transduction for baseline (T0), early APAP treatment time points (T30 min–24 h), and the endpoint (4 days APAP treatment and 21 days outgrowth) conditions. (**E**) Rank correlation p-values of median-normalized sgRNA read counts between treatment conditions.

HuH7-Cas9 was monoclonally selected and expression of Cas9 was confirmed by western blot (Fig. 1A, Supplementary Fig. 1). To determine the optimal dosage of APAP, HuH7-Cas9 cell count and viability were assessed daily (N = 3) in the presence of 0–20 mM APAP in growth media (Fig. 1B). A screening strategy was developed based on the rate of cell death in 15 mM APAP to assess the effect of the gene knockouts on cellular survival and proliferation with APAP treatment (Fig. 1C).

### CRISPR-Cas9 knock-out screen and deconvolution

HuH7-Cas9 cells (1.62 × 10^8^ total) were transduced with the lentiviral sgRNA library at an MOI of 0.5 resulting in >630x total library coverage at the time of transduction. The first replicate contains plasmid and samples collected at 0 h, 30 min, 3 h, 6 h, 12 h, 24 h, and 4d (end) of APAP treatment. The second replicate contains samples collected at 0, 24 h, and 4d of APAP treatment. A minimum of 2 × 10^7^ cells were collected per sample, resulting in 160x library coverage per sample as template for the 1^st^ PCR (Supplementary Fig. 2). The average library coverage of aligned reads calculated from amount of isolated DNA per sample was 205x and 284x, respectively for replicates 1 and 2. On average, 70% of the sequence reads aligned to the reference sgRNA library resulting in 230.9x average library coverage per replicate (Supplementary Table 1).

After 4 days of APAP treatment and 21 days outgrowth, the endpoint sample is significantly different from the plasmid library or T0 (p < 10^−10^) by comparison via Wilcoxon Rank-Sum test and there is a noticeable increase in variation of read counts after 4 days of drug treatment (Fig. 1D,E, Supplementary Table 2). Scatter plots of the read counts between the untreated and 24 h samples and the untreated and 4d samples show an increase in differential sgRNA count between 24 h and 4d of drug treatment (Supplementary Fig. 3a,b).

**Figure 2:**
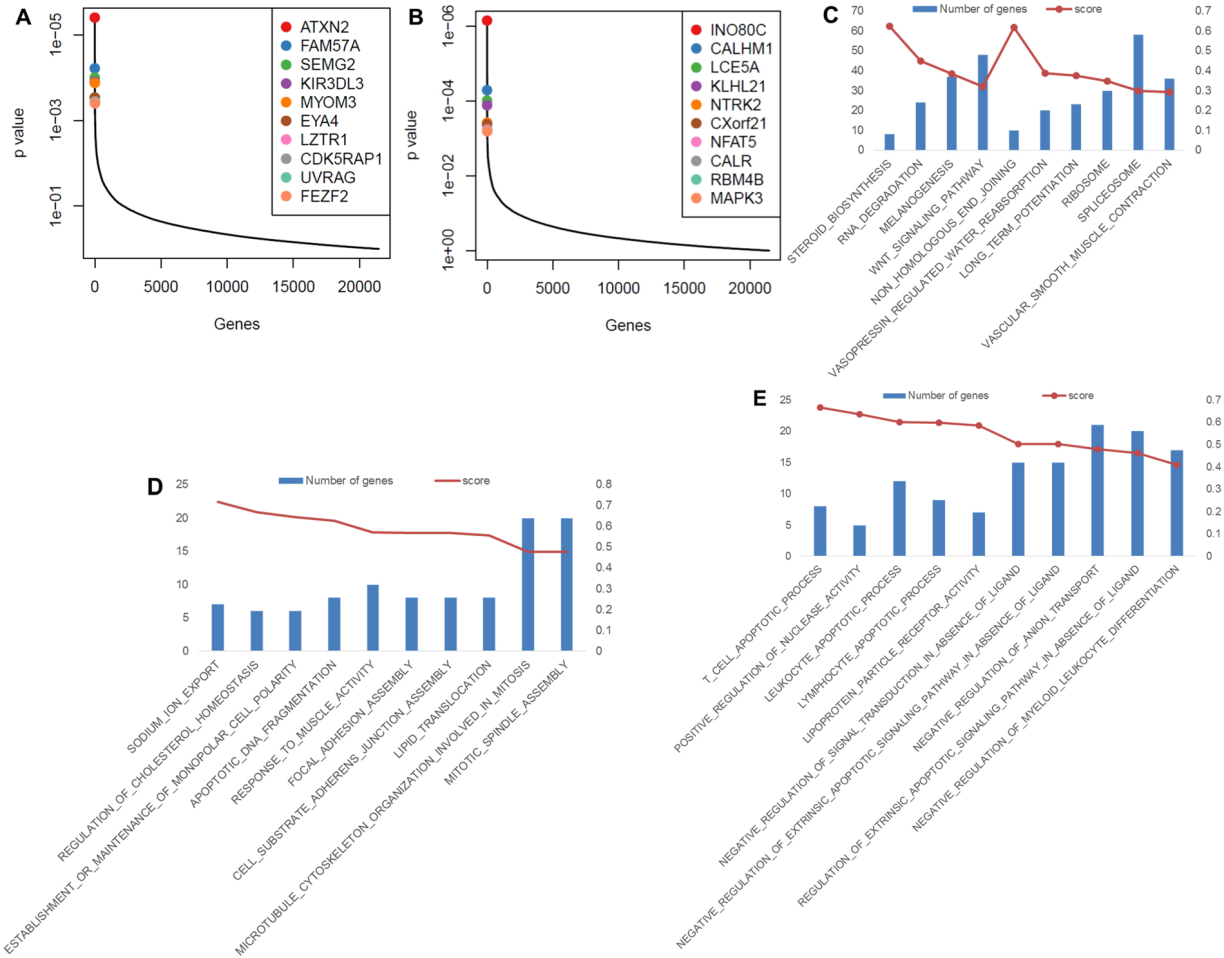
Positive and negative screening of response to APAP reveal top gene and pathway candidates. (**A**) Identification of top candidate genes using the p-values from positive RRA analysis of the 4d and T0 samples. Genes with the most positively selected sgRNAs are highlighted. (**B**) Identification of top candidate genes using the p-values from negative RRA analysis of the 4d and T0sample. Genes with the most negatively selected sgRNAs are highlighted. (**C**) Top 10 KEGG pathways negatively selected in the endpoint sample compared with the T0 sample. (**D**) Top 10 Gene Ontology pathways negatively selected in the endpoint sample compared with the T0 sample. (**E**) Top 10 Gene Ontology pathways positively selected in the endpoint sample compared with the T0 sample.

sgRNA read counts were analyzed to determine the gene-level and protein-level negative and positive screen rankings of individual time points and combined time points using RRA (Supplementary Data 1–8). The 4d (end) samples were compared with the untreated sample, revealing a number of genes containing sgRNA that are significantly decreased with APAP treatment (negatively selected, potentially essential) and significantly increased with APAP treatment (positively selected, potentially susceptible) (Fig. 2A,B). These gene knock-outs were significantly differentially expressed after 4d of APAP treatment represent a small population of cells remaining after most cells were killed by APAP. The ranked gene lists underwent GSEA pathway analysis against the All Gene Ontology and KEGG pathway gene sets, which returned statistically significant, highly ranked essential pathways in the negative screen analysis as well as a number of novel pathways in both the negative and positive screen analysis (Fig. 2C–E). Essential KEGG pathways are highly ranked in the negative screen after drug treatment, including *ribosome* and *spliceosome* pathways. Analysis of Gene Ontology pathways reveals other pathways important to cellular function are highly negatively selected and apoptotic processes are highly positively selected.

**Figure 3:**
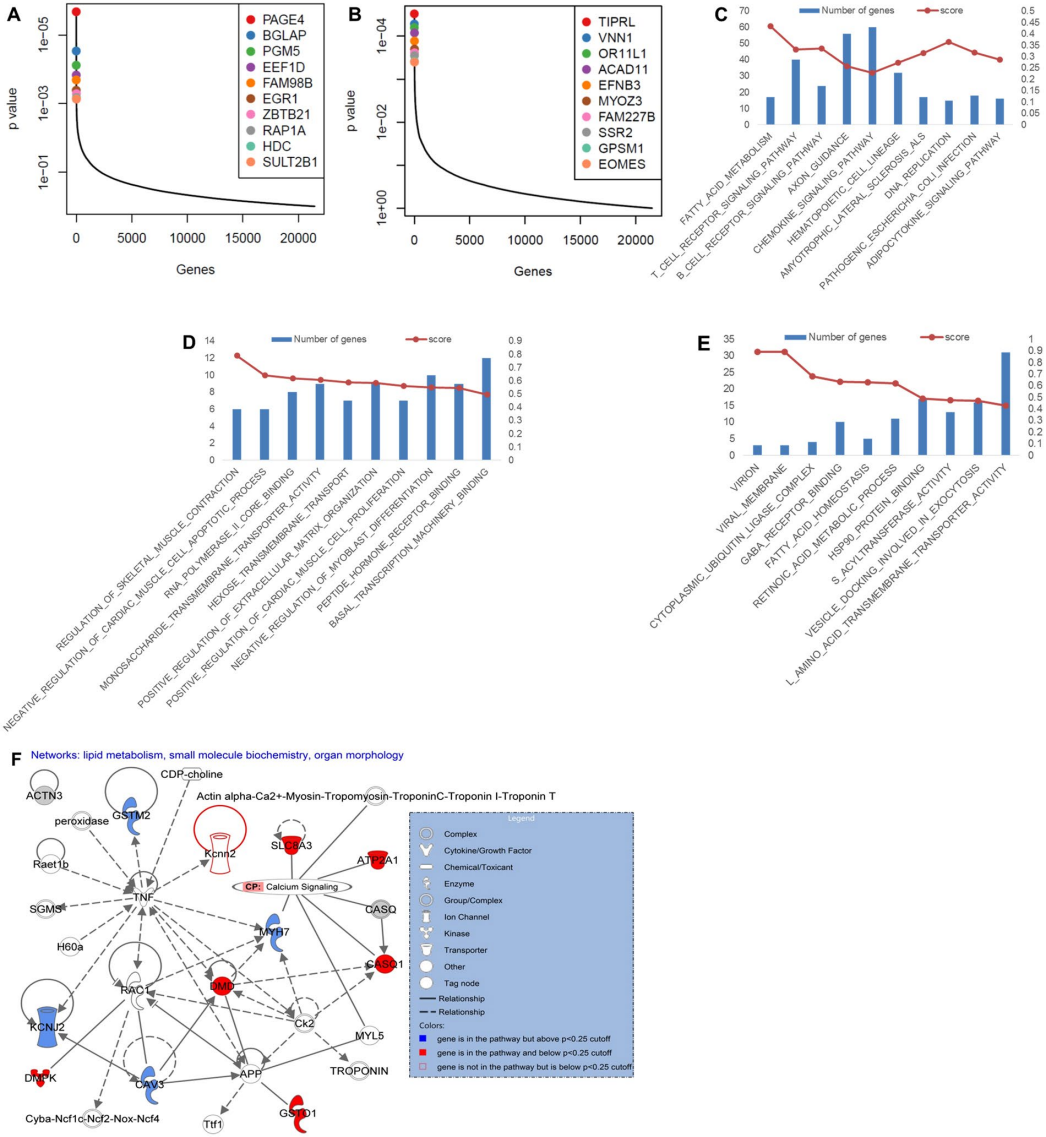
Highly ranked genes and pathways. (**A**) Identification of top candidate genes using the p-values from positive RRA analysis of the 24 h and T0 samples. Genes with the most positively selected sgRNAs are highlighted. (**B**) Identification of top candidate genes using the p-values from negative RRA analysis of the 24 h and T0 sample. Genes with the most negatively selected sgRNAs are highlighted. (**C**) Top 10 KEGG pathways negatively selected in the 24 h sample compared with the T0 sample. (**D**) Top 10 Gene Ontology pathways negatively selected in the 24 h sample compared with the T0 sample. (**E)** Top 10 Gene Ontology pathways positively selected in the 24 h sample compared with the T0 sample. (**F**) Top biological network identified by IPA from the top essential Gene Ontology pathway, regulation of skeletal muscle contraction, at 24 h of APAP treatment.

At 24 h APAP treatment, we observed a significantly different distribution of genes representing highly significant positive and negative changes in sgRNA expression (Fig. 3A,B). Pathway analysis by GSEA using the KEGG and Gene Ontology gene sets returned a number of novel pathways (Fig. 3C–E). The top negatively selected pathway after 24 hours of APAP treatment was *regulation of skeletal muscle contraction*. The top biological network identified from this pathway by Ingenuity Pathway Analysis (Qiagen) was *lipid metabolism*, *small molecule biochemistry*, *and organ morphology*, focusing around calcium signaling (Fig. 3F). We suspect this may be important to the injury introduced by the APAP overdose, and further study of genes involved in this calcium signaling identified from this screen (including *SLC8A3*, *ATP2A1*, *CASQ1*) are warranted. This correlates with existing literature, suggesting that calcium imbalance may affect APAP-induced hepatotoxicity^30,31^. Our data provide new and previously unrevealed targets for further experimentation.

We next sought to rank genes by time groups rather than specific time points with two main goals: 1) identify genes that are ranked highly (positive or negative) in early time points (30 min–24 h APAP exposure) vs. no treatment and 2) identify genes that are ranked highly (positive or negative) in all pooled APAP treated samples vs. no treatment. A literature search of the top 100 ranked genes (positively and negatively ranked, respectively) for each of these combinations of time points identified 44 unique genes (of 716 total unique genes queried) that are already associated with APAP and a vast majority which are novel (Table 1).

**Table 1:** The top 100 genes for various APAP time points were queried in pubmatrix to determine novelty.

PubMatrix	APAP	Acetaminophen	Hepatotoxic	Hepatotoxicity	Acute liver injury	Acute liver failure
24 h pos. top 100 genes × APAP	7	7	6	14	11	8
24 h neg. top 100 genes × APAP	5	5	5	5	5	5
4d pos. top 100 genes × APAP	7	6	6	8	6	8
4d neg. top 100 genes × APAP	7	7	2	8	8	4
all pos. top 100 genes × APAP	2	1	0	3	4	4
all neg. top 100 genes × APAP	6	6	4	7	6	4
30 min–24 h pos. top 100 genes × APAP	7	7	2	5	5	5
30 min–24 h neg. top 100 genes × APAP	6	6	4	7	5	5
genes in all 8 top 100 lists	800					
unique genes in all 8 top 100 lists	716					
unique genes with APAP hits	44(APAP), 42(acetaminophen)					

**Figure 4:**
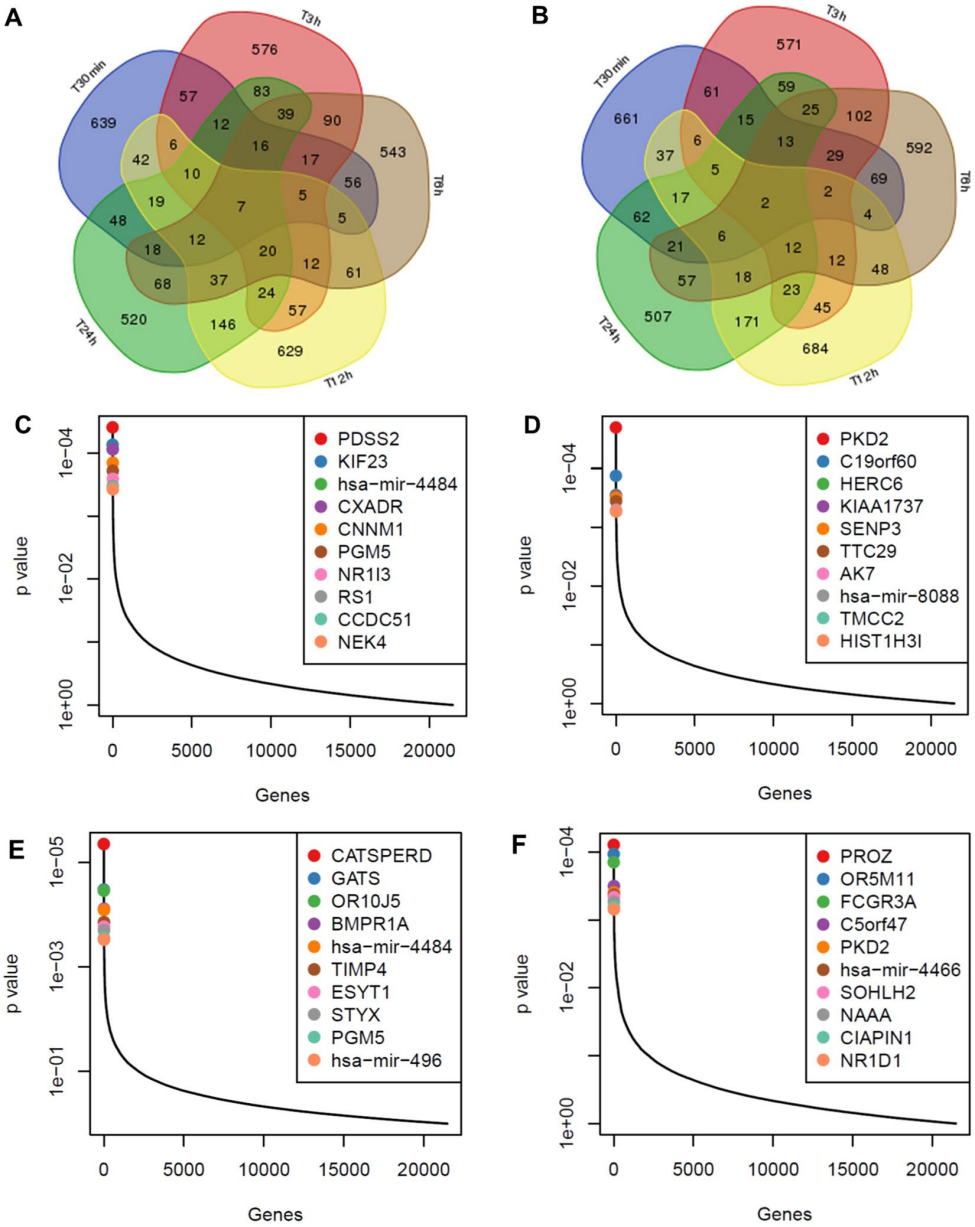
Identification of gene hits across the APAP time course in the CRISPR/Cas9 screen. (**A**,**B**) Venn diagrams of differently expressed genes in HuH7 cells treated with 15 mM APAP for 5 early time points. The diagrams show the number of gene knockouts significantly enriched by the treatment (**A**) and depleted by the treatment (**B**) for 5 time points (P < 0.05). The diagrams show the number of genes significantly modulated by the treatments. (**C**) Identification of top candidate genes using the p-values from positive RRA analysis based on all APAP time points vs. T0. Genes with the most positively selected sgRNAs are highlighted. (**D**) Identification of top candidate genes using the p-values from negative RRA analysis based on all APAP time points vs. T0. Genes with the most negatively selected sgRNAs are highlighted. (**E)** Identification of top candidate genes using the p-values from positive RRA analysis based on intermediate (30 min–24 h) APAP time points vs. T0. Genes with the most positively selected sgRNAs are highlighted. (**F**) Identification of top candidate genes using the p-values from negative RRA analysis based on intermediate (30 min–24 h) APAP time points vs. T0. Genes with the most negatively selected sgRNAs are highlighted.

We then grouped genes that were highly ranked at independent time points to isolate early and late acting genes. While a few genes contained sgRNA that are significantly enriched (or depleted) across all early time points, many are unique to the individual time points. While the sensitivity of the screen at very early times is likely lower than at later time points, early and late acting gene groups that are shared between time points or are unique to specific time points but represent statistically significant pathways may be important to drug response (Fig. 4A,B). To identify knocked-out genes which have global significance we compared all APAP-treated samples to the T0 samples (Fig. 4C,D). To identify knocked-out genes that were important for the early APAP response we compared the 30 min–24 h APAP treated samples to the T0 samples (Fig. 4E,F). These comparisons resulted in 5791 unique positively or negatively enriched significant genes (p < 0.05) in the combined 24 h APAP vs. T0, 4d APAP vs. T0, 30 min–24 h APAP vs. T0, or all APAP treatments vs. T0 gene rankings.

The RRA statistical method was chosen to rank gene knockouts because of its superior performance when compared with RSA and RIGER^32^. To validate our choice of statistical analysis method, we compared the Maximum Likelihood Estimate algorithm (MLE) to RRA, which has been shown to produce comparable gene ranking to RRA^33^. In a MLE analysis of all APAP time points compared with the T0 sample, 683 genes were statistically significant (p < 0.05), of which 442 (65%) were also statistically significant (p < 0.05) using the RRA method (v0.5.6) (Supplementary Data 9).

### Cross-referencing of other datasets

**Figure 5:**
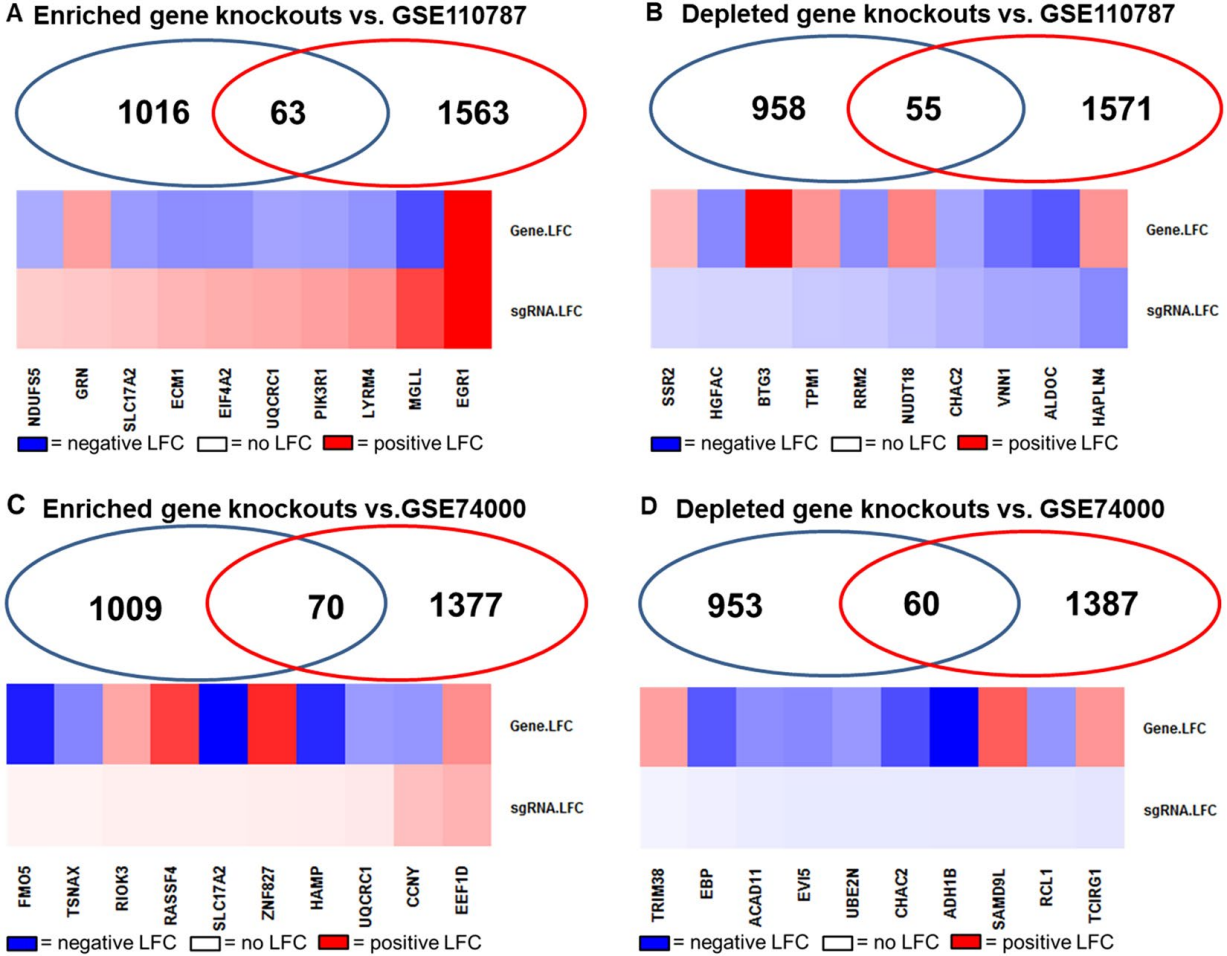
Validation of significant CRISPR/Cas9 screen hits by comparison with mouse ALI (GSE110787) and human ALF gene expression data (GSE74000). (**A**) Overlap of pos. CRISPR/Cas9 screen (p < 0.05, 24 h) with mouse RNA-Seq (p < 0.05, 24 h). Heat map of the log_2_ fold change of the most pos. selected sgRNAs (left to right). (**B**) Overlap of neg. CRISPR/Cas9 screen (p < 0.05, 24 h) with mouse RNA-Seq (p < 0.05, 24 h). Heat map of the differential log_2_ fold change of the most neg. selected sgRNAs (left to right). (**C**) Overlap of pos. CRISPR/Cas9 (p < 0.05, 24 h) with ALF microarray dataset GSE74000 (p < 0.05). Heat map of the differential log_2_ fold change of the most pos. selected sgRNAs (left to right). (**D**) Overlap of neg. CRISPR/Cas9 screen (p < 0.05, 24 h) with ALF microarray dataset GSE74000 (p < 0.05). Heat map of the differential log_2_ fold change of the most neg. selected sgRNAs (left to right).

In cuffdif, GSE110787 RNA-seq data from mice with and without APAP exposure were compared to assess the effect of APAP exposure on gene transcription. 1,626 of 46,073 gene probes had significantly differential gene expression after APAP exposure with an unadjusted p-val  < 0.05. 1,025 genes have − log_2_ fold change with p < 0.05 and 601 genes have +log_2_ fold change with P < 0.05 (Supplementary Fig. 4a, Data 10). Overlap between the genes that are highly ranked in the CRISPR screen at 24 h APAP treatment (2,082 gene knockouts, p < 0.05) and GSE110787 (p < 0.05) warrant validation *in vivo*. (Fig. 5A,B).Overall, 63 enriched gene knockouts and 55 depleted gene knockouts (24 h, p < 0.05) overlap with the significantly differentially expressed genes in the mouse model of ALF after 24 h drug treatment.

Secondary data from human sources was used to cross-validate the CRISPR screen findings. In GEO2R, microarray data from 3 APAP-induced ALF liver samples were compared to 2 healthy liver samples (GSE74000). 1,679 of 54,675 probes have an FDR-adjusted p-value of <0.05. 1,251 probes have − log_2_ fold change with p < 0.05 and 428 probes have +log_2_ fold change with p < 0.05 (Supplementary Fig. 4b). We compared genes with p < 0.05 to genes that were significantly enriched and depleted in our CRISPR screen (p < 0.05) to identify overlap and ascertain the relationship between sgRNA depletion or enrichment and gene expression (Fig. 5C,D). Overall, 63 enriched gene knockouts and 55 depleted gene knockouts (24 p < 0.05) overlap with the significantly differentially expressed genes in the human ALF data.

**Figure 6:**
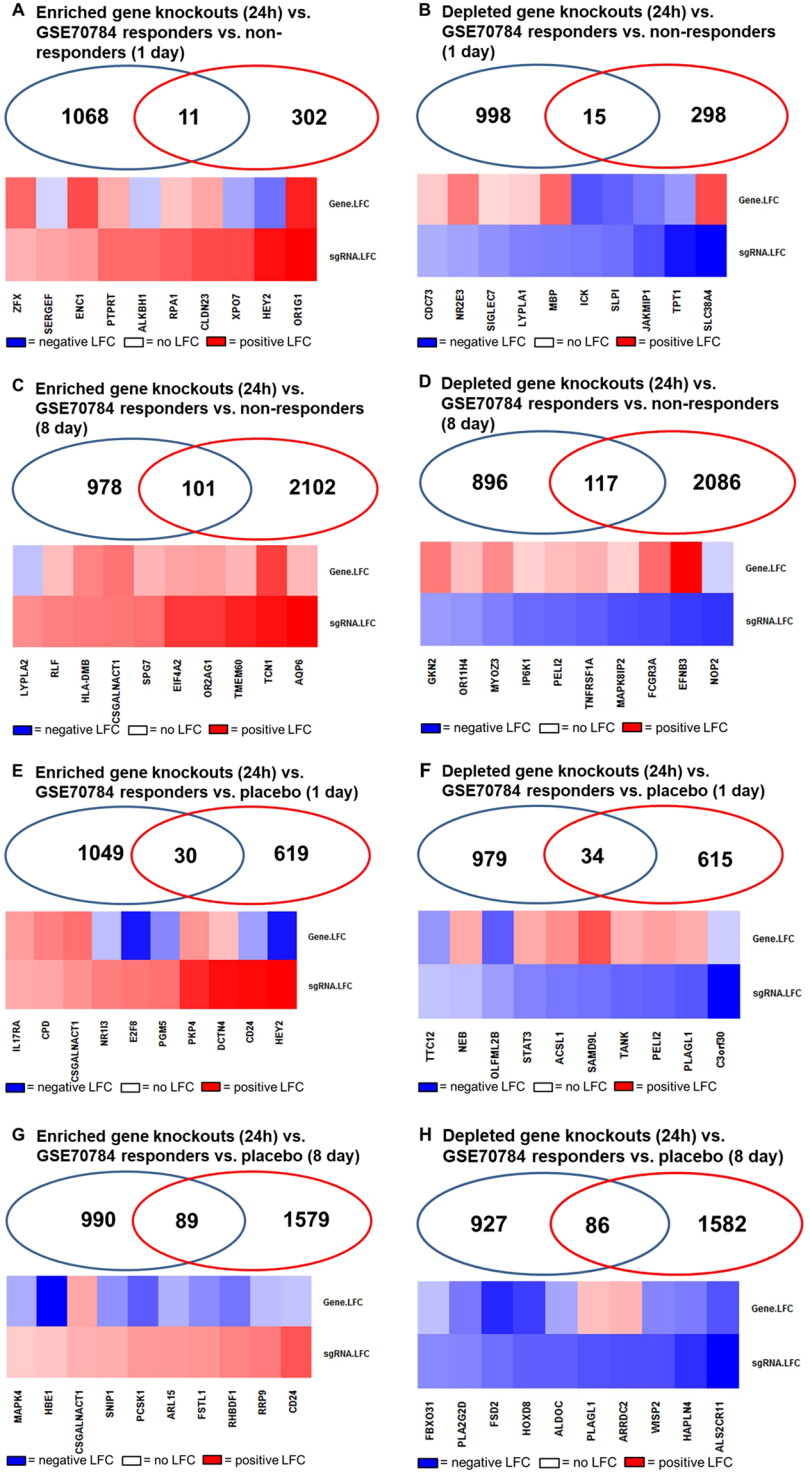
Validation of significant CRISPR/Cas9 screen hits by comparison with human gene expression data (GSE70784). (**A**) Overlap of pos. CRISPR/Cas9 screen (p < 0.05, 24 h) with APAP overdose microarray dataset GSE70784 responders vs. Non-responders (1 day, p < 0.05). Heat map of differential log_2_ fold change of the most pos. selected sgRNAs (left to right). (**B**) Overlap of neg. CRISPR/Cas9 screen (p < 0.05, 24 h) with APAP overdose microarray dataset GSE70784 responders vs. Non-responders (1 day, p < 0.05). Heat map of differential log_2_ fold change of the most neg. selected sgRNAs (left to right). (**C**) Overlap of pos. CRISPR/Cas9 screen (p < 0.05, 24 h) with APAP overdose microarray dataset GSE70784 responders vs. Non-responders (8 days, p < 0.05). Heat map of differential log_2_ fold change of the most pos. selected sgRNAs (left to right). (**D**) Overlap of neg. CRISPR/Cas9 screen (p < 0.05, 24 h) with APAP overdose microarray dataset GSE70784 responders vs. Non-responders (8 days, p < 0.05). Heat map of differential log_2_ fold change of the most neg. selected sgRNAs (left to right). **(E**) Overlap of pos. CRISPR/Cas9 screen (p < 0.05, 24 h) with APAP overdose microarray dataset GSE70784 responders vs. Placebo (1 day, p < 0.05). Heatmap of differential log_2_ fold change of the most pos. selected sgRNAs (left to right). (**F**) Overlap of neg. CRISPR/Cas9 screen (p < 0.05, 24 h) with APAP overdose microarray dataset GSE70784 responders vs. Placebo (1 day, p < 0.05). Heatmap of differential log_2_ fold change of the most neg. selected sgRNAs (left to right). (**G**) Overlap of pos. CRISPR/Cas9 screen (p < 0.05, 24 h) with APAP overdose microarray dataset GSE70784 responders vs. Placebo (8 days) (p < 0.05). Heatmap of differential log_2_ fold change of the top 10 genes with the most pos. selected sgRNAs (left to right). (**H**) Overlap of neg. CRISPR/Cas9 screen (p < 0.05, 24 h) with APAP overdose microarray dataset GSE70784 responders vs. Placebo (8 days) (p < 0.05). Heatmap of differential log_2_ fold change of the most neg. selected sgRNAs (left to right).

A second dataset, GSE70748, was chosen to filter genes identified in the CRISPR screen that have also been identified in blood in humans who have been dosed with APAP. In GEO2R, microarray data from 12 APAP responder blood samples were compared to 32 non-responders using days 1 and 8 independently (GSE70784). No probes had an FDR-adjusted p-val < 0.05, so the unadjusted p-values were referenced. After 1 day of APAP dosing 362 of 20,173 probes have an unadjusted p-val  < 0.05, of which 148 probes have −log_2_ fold change with p < 0.05 and 214 probes have +log_2_ fold change with P < 0.05 (Supplementary Fig. 5a). After 8 days of APAP dosing 2445 of 20,173 probes had an unadjusted p-val < 0.05, of which 314 probes have − log_2_ fold change with p < 0.05 and 2,131 probes have + log_2_ fold change with P < 0.05 (Supplementary Fig. 5b). We compared genes with p < 0.05 to genes that were significantly enriched and depleted in our CRISPR screen (p < 0.05) to identify overlap and ascertain the relationship between sgRNA depletion or enrichment and gene expression at 24 h APAP treatment (Fig. 6A–D). Overall, 11 enriched gene knockouts and 15 depleted gene knockouts (24 h, p < 0.05) overlap with the significantly differentially expressed genes in non-acute overdose (drug responders vs. non-responders) after 1d of exposure. 101 enriched CRISPR gene knockouts and 117 depleted gene knockouts (24 h, p < 0.05 overlap with the significantly differentially expressed genes between drug responders and non-responders after 8d of exposure.

Using the same GSE70784 dataset in GEO2R, microarray data from 12 APAP responder blood samples were compared to 10 placebo controls using days 1 and 8 independently. After 1 day of APAP dosing 697 of 20,173 probes had an unadjusted p-val < 0.05. Of these, 244 probes have − log_2_ fold change with p < 0.05 and 453 probes have +log_2_ fold change with P < 0.05 (Supplementary Fig. 5c). After 8 days of APAP dosing 1,801 of 20,173 probes had an unadjusted p-val < 0.05, of which 1248 probes have − log_2_ fold change with p < 0.05 and 553 probes have +log_2_ fold change with P < 0.05 (Supplementary Fig. 5d). We compared genes with p < 0.05 to genes that were significantly enriched and depleted in our CRISPR screen (p < 0.05) to identify overlap and ascertain the relationship between sgRNA depletion or enrichment and gene expression at 24 h APAP treatment (Fig. 6E–H). 30 enriched gene knockouts and 34 depleted gene knockouts (24 h, p < 0.05) overlap with the significantly differentially expressed genes in non-acute overdose (responders vs. placebo) after 1d of exposure. 89 enriched CRISPR gene knockouts and 86 depleted gene knockouts (24 h, p < 0.05 overlap with the significantly differentially expressed genes in non-acute overdose after 8d of exposure.

Of the genes overlapping the CRISPR screen at 24 h APAP exposure (p < 0.05) and 1d APAP exposure vs. placebo in GSE70784, 7 up regulated genes and 8 downregulated genes remain significantly up or down regulated after 8d APAP treatment (GSE70784, p < 0.05). These overlaps rise to 10 and 20 genes, respectively, when the CRISPR gene knockout list is expanded to include all significant gene knockouts across all treatment times. Similarly, 6 downregulated genes remain significantly down regulated after 8d APAP treatment when the CRISPR overlapping APAP responders are compared with non-responders (GSE70784, p < 0.05). 13 genes are downregulated when the CRISPR gene knockout list is expanded to include all significant gene knockouts across all treatment times. Overall, our CRISPR screen data best overlaps the long-term exposure (8d). We additionally observe that there is little overlap between the differentially expressed genes in the early (1d) and late (8d) chronic exposure data of GSE70784 when filtered by gene knockouts that are significantly enriched or depleted in the CRIPSR screen. This suggests a dramatic shift in gene expression between early and longer-term exposure. We also observe better overlap when we include significant gene knockouts from other time points observed from the CRISPR screen.

We then isolated only genes (or gene knockouts in the case of the CRISPR screen) that were significantly differentially expressed across the CRISPR, mouse, and human studies. 523 genes (369 unique, 6% of CRISPR-Cas9 screen genes with p < 0.05) overlap the mouse RNA-seq and CRISPR “top lists” (4d, 24 h, Int, and All, p < 0.05, representing 5,791 unique genes with significant enrichment or depletion in the CRISPR screen). 57 of the 67 unique genes overlapping CRISPR, mouse, and GSE74000 p < 0.05 lists (0.1% of CRISPR-Cas9 screen genes with p < 0.05) are not previously reported to have a role in APAP metabolism, and 51/67 have consistent expression in mouse and GSE74000 and within CRISPR lists. When we compare the GSE70784 1 day responder vs. placebo to the CRISPR and mouse RNA-seq datasets, 12 of the 16 overlapping unique genes are novel (0.3% of CRISPR-Cas9 screen genes with p < 0.05, p < 0.05 overlap the main CRISPR analyses and the mouse RNA-seq) and 10 of the 16 have consistent expression between CRISPR analysis or between gene expression dataset. When we compare the GSE70784 8 day responder vs. placebo to CRISPR and Mouse datasets 36 of the 38 overlapping unique genes are novel (0.7% of CRISPR-Cas9 screen genes with p < 0.05, p < 0.05 overlap the main CRISPR analyses and the mouse RNA-seq) and 22 of the 38 have consistent expression between CRISPR analysis or between gene expression dataset. The largest number of genes overlapping with the CRISPR-Cas9 screen data was observed with the GSE70784 8d day responder vs. non-responder and responder vs. placebo datasets. (Supplementary Table 3). A number of the genes which had statistically significant differential expression in the *in vivo* datasets have known relationships with APAP (top 100 genes per data set), although as previously seen with the CRISPR screen, many are novel findings (Supplementary Table 4). These candidates which show consistent and significant differential expression in ALI (GSE70784) and ALF (mouse RNA-seq and GSE74000) and whose knockout impacts survival of APAP overdose need further study to evaluate the mechanisms and pathways by which they function.

We suspect that NAD metabolism may play an important role in survival of acetaminophen injury and to this end we identified a number of genes involved in NAD metabolism which are also highly ranked in the CRISPR screen time points. A list of 48 genes identified based on Nikiforov *et al*., 2015 was compared with statistically significant CRISPR hits (p < 0.05)^34^. We identified 9 NAD metabolism in our screen data (Supplementary Table 5). Additionally, data from our lab suggest overexpression of NAMPT, a gene involved in NAD salvage, is protective against APAP-induced hepatotoxicity *in vivo*^35^.

We considered genes for functional validation which were in the top 10 of a CRISPR list and were also significantly differentially expressed in the GEO or mouse RNA-seq datasets (p < 0.05), with a preference for genes with a p < 0.05 in multiple positive or negative ranked lists. Novelty was assessed by literature search and essentiality was determined from essentialgene.org. A number of genes that were highly ranked in the CRISPR screen (positive or negative), and overlapped with other gene sets (human and mouse gene expression with and without APAP, p < 0.05), are identified as essential genes (essentialgene.org). These genes include *PGM5*, *KIF23*, *C19orf60*, *BMPR1A*, *PDSS2*, *CXADR*, *SSR2*, *TMCC2*, *RDH13*, *and EGR1* (Supplementary Data 11). Additional genes that were highly ranked in the CRISPR screen, and overlapped with the other gene sets (human and mouse gene expression with and without APAP), have previously published relationships with APAP metabolism (pubmatrix.irp.nia.nih.gov). These genes include *EGR1*, *VNN1*, *NR1I3*. Genes ranked highly in both our screen and previous publications support the selection method used to filter candidate genes. Novel, non-essential genes identified by this study for further study include *LZTR1*, *NAAA*, *ATG2B*, *MYOZ3*, *EFNB3*, *OR5M11*, *FCGR3A*, *PROZ*, *EEF1D*, *ACAD11*, and *TMCC2* (Supplementary Data 11). These genes are pathogenic (positively ranked) or protective (negatively ranked) and have potential for utility in development of diagnostic, risk-assessment, or therapeutic biomarkers.

### Genes containing significant APAP SNPs

133 gene names were identified from the literature as nearest-neighbors or containing 147 APAP injury-associated single nucleotide polymorphisms (SNPs)^36^. 22 of the genes were significantly enriched or depleted in the screen time points (Supplementary Table 6).

### Drug-gene interactions of top candidate genes

Further analysis of top candidate genes described in this study (Supplementary Data 11, Tables 5–6) identified a number of candidate genes that may be suitable for re-purposing to treat APAP-induced hepatotoxicity. Of the 54 unique candidate genes that were analyzed, 153 drug-gene interactions were identified for 19 genes (Supplementary Data 12). Of these, 14 genes were annotated with drug-gene interactions of known effects (Table 2). Notably, 3 novel genes are targets of existing drugs, which may be suitable re-purposed therapeutics against APAP-induced hepatotoxicity. *BMPR1A*, identified as a susceptible gene by the CRISPR-Cas9 screen, is inhibited by CHEMBL3186227. *PROZ*, identified as a protective gene by the CRISPR-Cas9 screen, is activated by Menadione. *HSD11B1*, a gene that was susceptible in the CRISPR-Cas9 screen, is inhibited by Carbenoloxone, CHEMBL222670, CHEMBL2153191, CHEMBL2177609, and Phenylarsine Oxide. An additional 3 genes, *NR1I3*, *SIRT3*, and *GSTP1*, have known roles in APAP hepatotoxicity that were correctly predicted by our CRIPSR-Cas9 screen and are targets of existing drugs that may be suitable for re-purposing^37–39^. These 6 genes are excellent candidate targets for re-purposing existing drugs to treat APAP-induced ALI and ALF. An additional 3 genes, *SIRT1*, *GPX4*, and *GSS*, were identified as targets of drugs with known gene interactions, however the CRISPR-Cas9 screen did not agree with the published gene role (protective or susceptible) in APAP-induced hepatotoxicity^40–42^.

**Table 2:** Top candidate genes with known drug effects annotated by the DRUG Gene Interaction Database (www.dgidb.org).

Gene	Gene Effect on ALF	Known Drug	Drug Effect	Drug Effect matches Gene Effect?
*BMPR1A*	susceptible (CRISPR screen)	CHEMBL3186227	inhibitor	yes
*FCGR3A*	protective (CRISPR screen)	GLOBULIN, IMMUNE	antagonist	no
*NAAA*	protective (CRISPR screen)	CARBENOXOLONE	inhibitor	no
*NAAA*	protective (CRISPR screen)	FLUFENAMIC ACID	inhibitor	no
*NR1I3*	susceptible (PMID: 12376703, and CRISPR screen)	PRASTERONE	activator	no
*NR1I3*	susceptible (PMID: 12376703, and CRISPR screen)	CHEMBL458603	agonist	no
*NR1I3*	susceptible (PMID: 12376703, and CRISPR screen)	CLOTRIMAZOLE	antagonist	yes
*NR1I3*	susceptible (PMID: 12376703, and CRISPR screen)	MECLIZINE	antagonist modulator	yes
*PROZ*	protective (CRIPSR screen)	MENADIONE	activator	yes
*HSD11B1*	susceptible (CRISPR screen)	CARBENOXOLONE	inhibitor	yes
*HSD11B1*	susceptible (CRISPR screen)	CHEMBL222670	inhibitor	yes
*HSD11B1*	susceptible (CRISPR screen)	CHEMBL2153191	inhibitor	yes
*HSD11B1*	susceptible (CRISPR screen)	CHEMBL2177609	inhibitor	yes
*HSD11B1*	susceptible (CRISPR screen)	PHENYLARSINE OXIDE	inhibitor	yes
*HSD11B1*	susceptible (CRISPR screen)	PREDNISONE	ligand	unknown
*SIRT1*	protective (PMID 29084443), susceptible (CRISPR screen)	CHEMBL257991	activator	unknown
*SIRT1*	protective (PMID 29084443), susceptible (CRISPR screen)	SODIUM LAURYL SULFATE	inhibitor	unknown
*SIRT1*	protective (PMID 29084443), susceptible (CRISPR screen)	CHEMBL420311	inhibitor	unknown
*SIRT1*	protective (PMID 29084443), susceptible (CRISPR screen)	SPLITOMICIN	inhibitor	unknown
*SIRT3*	susceptible (PMID 21720390, CRISPR screen)	SODIUM LAURYL SULFATE	inhibitor	yes
*GPX2*	protective (CRISPR screen)	GLUTATHIONE	cofactor	unknown
*GPX4*	protective (PMID 25962350), susceptible (CRISPR screen)	GLUTATHIONE	cofactor	unknown
*GSS*	protective (PMID 11287661), susceptible (CRISPR screen)	ACETYLCYSTEINE	stimulator	no
*GSTP1*	susceptible (PMID 11058152; CRIPSR screen)	EZATIOSTAT HYDROCHLORIDE	inhibitor	yes
*KCNJ3*	protective (CRISPR 4d), susceptible (CRISPR all APAP samples)	CHEMBL2409106	activator	unknown
*KCNJ3*	protective (CRISPR 4d), susceptible (CRISPR all APAP samples)	CHEMBL116590	channel blocker	unknown
*KCNJ3*	protective (CRISPR 4d), susceptible (CRISPR all APAP samples)	HALOTHANE	inhibitor	unknown
*NAMPT*	protective (PMID 29684358)	TEGLARINAD CHLORIDE	inhibitor	no

### Functional validations of candidate genes

**Figure 7:**
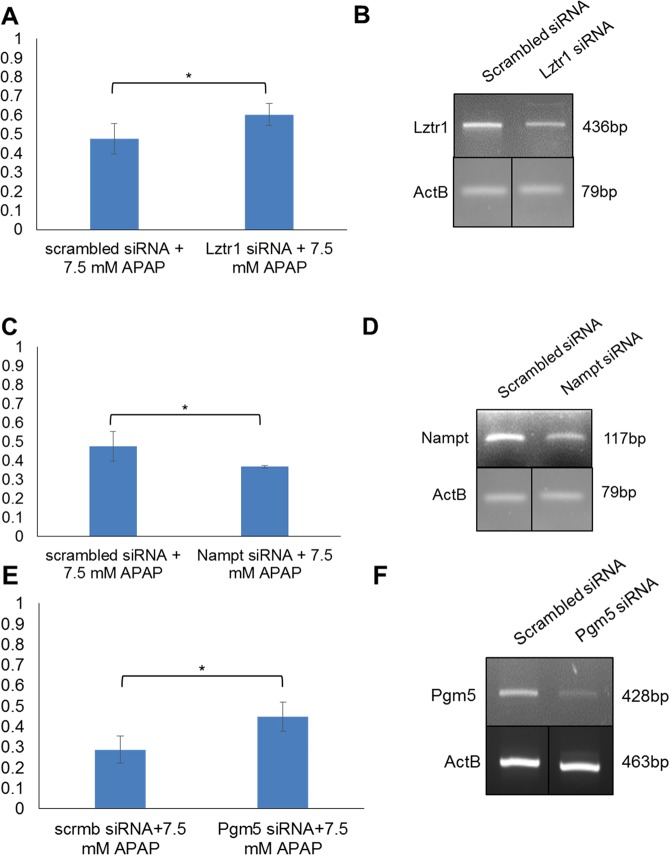
Validation experiments in primary mouse hepatocytes. (**A**) Viability of primary mouse hepatocytes transfected with 25 Mm scrambled or *Lztr1* siRNA after treatment with 7.5 mM APAP for 3 h, normalized to *Lztr1* siRNA transfected, untreated cells, measured by luminescent ATP assay. N = 4 and error bars represent standard deviation. *p < 0.05. (**B**) sqPCR from cDNA prepared from RNA collected 25 h post-transfection with 25 Mm scrambled or *Lztr1* siRNA. *ActB* qPCR reactions were conducted concurrently, run on the same gel in non-adjacent lanes, and imaged concurrently. (**C**) Viability of primary mouse hepatocytes transfected with 25 Mm scrambled or *Nampt* siRNA after treatment with 7.5 mM APAP for 3 h, normalized to *Nampt* siRNA transfected, untreated cells, measured by luminescent ATP assay. N = 4 and error bars represent standard deviation. *p < 0.05. (**D**) sqPCR from cDNA prepared from RNA collected 25 h post-transfection with 25 Mm scrambled or Nampt siRNA. *ActB* qPCR reactions were conducted concurrently, run on the same gel in non-adjacent lanes, and imaged concurrently. (**E**) Viability of primary mouse hepatocytes transfected with 50–100 Mm scrambled or *Pgm5* siRNA after treatment with 7.5 mM APAP for 3 h, normalized to *Pgm5* siRNA transfected, untreated cells, measured by luminescent ATP assay. N = 4 and error bars represent standard deviation. *p < 0.05. (**F**) sqPCR from cDNA prepared from RNA collected 25 h post-transfection with 50 Mm scrambled or *Pgm5* siRNA. *ActB* qPCR reactions were conducted concurrently using the *ActB* F2 and R2 primers, run on the same gel in non-adjacent lanes, and imaged concurrently. Full-length gels are presented in Supplementary Fig. 6.

Mouse *Lztr1*, Nampt, and *Pgm5* were selected for further *in vitro* validations of their functional effect of survival of APAP injury in primary mouse hepatocytes. *Nampt* knockdown by siRNA was significantly pathogenic when compared with a scramble control after 3 h APAP treatment (Fig. 7A,B, Supplementary Fig. 6a). *Lztr1* knockdown by siRNA was significantly protective when compared with a scramble control after 3 h APAP treatment (Fig. 7C,D, Supplementary Fig. 6b). *Pgm5* knockdown by siRNA resulted in a significant increase in cellular survival after 3 h of APAP treatment when compared with the scrambled control (Fig. 7E,F, Supplementary Fig. 6c).

## Discussion

This study has identified a number of novel and previously unrevealed regulators of APAP-induced hepatotoxicity by employing state of the art genome-wide CRISPR-Cas9 screen in a hepatocyte cell line. Selected targets have been validated in primary hepatocytes and cross-referenced in other available data sets of human and mouse involvement. Our study has illustrated the power of a genome-wide CRISPR-Cas9 screen to systematically identify novel genes involved in APAP induced hepatocyte toxicity and most importantly, it provide a rich resources for further experimentation to identify potential new diagnostic targets or to develop novel therapeutic modalities to APAP induced hepatocyte toxicity.

Validation of the screen findings was sought at multiple steps in the analysis and by siRNA in primary hepatocytes. Inspection of the significant genes revealed overlap with human microarray and mouse RNA-seq studies of APAP overdose. Additionally, several top genes identified from the screen for further study already had known associations with APAP in the literature. Lastly, some of the genes identified from the screen for further study have been previously identified as essential. While these genes were not essential in our study, their relationship with APAP treatment would support their roles in critical cellular functions that, when disrupted, result in cell death.

Although few genes were completely removed from the pooled mutant cell population prior to APAP treatment, thousands were missing after 4 days of APAP treatment. Based on the kill curve 4 days of APAP treatment results in about 1% surviving cells, indicating a majority of the cells being killed. The survival of cells with low numbers of sgRNAs is only statistically important if the proportion within the surviving population is significantly different than the starting population consistently across multiple sgRNAs per gene. The early time points (30 min to 24 h) in this screen are base off of traditional gene expression screening techniques. By considering the impact of drug selection at early time points we can better assess the early and late response genes involved in drug toxicity. We propose that a Wilcoxon Rank-Sum value of p < 10^−10^ may be too stringent for addressing finer scale effects of gene knockout.

Using GSEA pathway analysis our screen identified WNT signaling (KEGG pathway) as a very strongly depleted pathway and also identified positive regulation of Notch Signaling (All Gene Ontology gene set) as a significantly depleted pathway (p < 0.05). Notch signaling has been previously identified as essential to survival of APAP^43^. To further validate our screening methodology, both spliceosome and ribosome KEGG pathways are among the most strongly depleted pathways after 4 days of APAP treatment. Our top negatively selected pathway after 24 h APAP treatment, *regulation of skeletal muscle contraction*, corroborated existing work, suggesting that intracellular calcium may be important to response to APAP. However, the role of this pathway in APAP-induced hepatotoxicity is unclear.

The 3 gene expression datasets all used distinct sampling methodologies, when combined with the CRISPR-Cas9 screen data, produced a comprehensive picture of changes in gene expression after APAP overdose. GSE70784 consists of blood samples from participants that are dosed with the daily maximum of APAP daily for an extended time. These data reflect a more chronic drug exposure, and response to the drug is measured by ALT. GSE74000 consisted of liver biopsies from Livers being replaced after APAP-induced ALF and liver biopsies obtained from non-ALF donors. This dataset, although it contains few samples, represents differential gene expression in humans at the 4d-point of the disease. The mouse RNA-seq data GSE110787 provided an extremely controlled population with controlled APAP dosage, avoiding issues of inter-population variabilities that may affect studies in human populations.

The local inflammatory response and accumulation of neutrophils, which is not considered necessary to the initiation of progression of ALF contribute a major role in clearing necrotic cells and alter the liver injury micro-environment. In addition the inflammasome contributes greatly to the late stage of injury with activation of caspase-1 and IL1β with further cytokines and chemokines contributing to the recruitment of neutrophils and monocytes^44^. This late-stage of injury would be better captured by the mouse RNA-seq (ALF, GSE110787) and human microarray (ALF, GSE74000) datasets, since they represent a late-stage disease in a whole organism, which includes inflammatory and immune interactions not present in hepatocytes alone. It is therefore unsurprising that we observed the best overlap of the CRISPR screen data with the human liver injury microarray data (GSE70784).

This approach addresses APAP-induced liver injury in 2 distinct ways. First, we identified genes with a role in APAP metabolism by assessing the effect of gene knockouts on cell proliferation and survival. Next, we identified genes that were differentially expressed in response to APAP. The combination helps us to build hypotheses about the role of these genes in the disease process. This cross-validation with other APAP datasets is targeted at identifying genes that are important to APAP metabolism and may be novel diagnostic or therapeutic biomarkers. Genes that are highly ranked in the CRISPR screen (p < 0.05) and whose RNA are expressed differentially at high enough levels that a blood sample (preferable) or liver biopsy (less preferable) could be used to detect changes in expression levels resultant from APAP overdose rapidly in clinic. Novel genes identified by this method that were highly ranked in the CRIPSR-Cas9 screen and in the gene expression data are the strongest candidates for further study.

We tested the effect of siRNA knockdown of *Lztr1*, *Nampt*, *Pgm5*, and *Naaa* in primary mouse hepatocytes to validate our screen findings. We demonstrate that Leucine Zipper Like Transcription Regulator 1 (*LZTR1*) knockout in HuH7 and knockdown in mouse cells increase cellular survival of APAP-induced injury. *LZTR1* has a positive LFC in the APAP-exposed human microarray data GSE70784, suggesting that the while the gene knockout increases survival of APAP, it is also elevated in APAP-treated subjects (Supplementary Data 11). *LZTR1* mutations are associated with Noonan Syndrome 10, Schwannomatosis-2, gastric cancer, ventricular septal defects, and deletion of the gene may be associated with DiGeorge syndrome^45–49^. The GO annotations for *LZTR1* include transcription factor activity and sequence-specific DNA binding. The protein localizes to the golgi, where it is thought to have a stabilizing effect.

Nicotinamide Phosphoribosyltransferase (*NAMPT*, PDBID 4LVF.A) was selected for further study because although it is not significant in this screen, other lab data demonstrates a protective effect of overexpression against APAP-induced hepatotoxicity. In mice, *Nampt* has reduced expression after APAP treatment (LFC = −0.476, p < 0.05). This in combination with the number of other NAD metabolism genes that are significantly ranked in this screen led us to validate the observed effect of *NAMPT* knockout in HuH7 with knockdown in mouse hepatocytes, which we found to increase susceptibility to APAP-induced injury. NAMPT protein is involved in the catalysis of the biosynthesis of the nicatinomide adenine dinucleotide. NAMPT’s role in NAD salvage is thought to be important to a number of metabolism and aging-related conditions^50–57^. It is involved in the NAD metabolism and Common Cytokine Receptor Gamma-Chain Family Signaling pathways. GO annotations include protein homodimerization activity and drug binding. NAMPT’s role in APAP-induced hepatotoxicity does however need further study in whole organisms to evaluate its role during the different stages of liver injury. The secreted form of Nampt functions as both a cytokine and adipokine and functions to inhibit neutrophil apoptosis which is implicated in the second phase of acetaminophen-induced injury^58^.

Phosphoglucomutase 5 (*PGM5*) knockdown increased cellular survival of APAP treatment, validating our CRISPR/Cas9 screen finding that knockout of the gene is protective (Supplementary Data 11). *PGM5* has a negative LFC in the APAP-exposed human microarray data GSE70784, suggesting that the gene knockout increases survival of APAP exposure and gene expression is decreased after APAP exposure. PGM5 does not exhibit phosphoglucomutase activity and is a component of cell-cell and cell-matrix junctions. It is expressed at high levels in smooth muscle and is essential in the metabolism of galactose and glycogen and is involved in the Porphyrin and chlorophyll metabolism pathway. GO annotations include structural molecule activity, intramolecular transferase activity, and phosphotransferase activity. Abnormal expression and mutation of *PGM5* are associated with a number of diseases, including Duchenne’s Muscular Dystrophy and colorectal tumorigenesis^59,60^.

Although we were able to confirm knockdown of mouse *Naaa in vitro*, we were not able to validate the increase in susceptibility observed in the CRISPR-CAS9 screen. It is possible that the effect was too small in the conditions used for the validation experiments, or that a true knockout is needed to observe the effect.

It is widely accepted that the cytochrome P450 isoform play an important role in APAP metabolism. While we expected to see the cytochrome P450 isoforms higher in the gene rankings of the negative screen, it is unsurprising that they are not highly ranked. It is suspected that multiple isoforms can regulate the metabolism of APAP, so it is possible that others are compensating for the knocked out isoform. The low, though not totally absent, expression of some CYPs in HuH7 arguably increases the potential for this system to reveal non-canonical mechanisms of survival and susceptibility^61^. HuH7 additionally metabolized NAPQI by glucuronidation and sulfation at low levels^7,61^. Although there are always concerns when using a cell line to study a biological mechanism, HuH7 has been used successfully for studies of drug metabolism^61,62^. To carry out the CRISPR-Cas9 screen it was necessary to use a cell line that could be transduced and didn’t require differentiation. Whenever possible, we validated our findings in primary mouse hepatocytes.

To better control for potential differences in drug metabolism across systems and to identify the most promising candidate genes, the CRIPSR-Cas9 gene knockout rankings were cross-referenced with multiple human and mouse datasets to select the most promising candidate genes. We also identified genes with likely and known associations with APAP-induced hepatotoxicity (NAD metabolism and genes containing polymorphisms). Further study of the polymorphisms in these genes could result in a diagnostic or prognostics SNP panel. Further study of the role of these genes could inform their use in targeted therapies. These candidate genes were assessed for drugability by existing drugs as a means to more quickly bring forward new therapies. Indeed, 6 candidate genes (3 novel and 3 known) are targets for existing drugs which have an interaction predicted to be protective against APAP-induced hepatotoxicity.

## Conclusions

Collectively, this study has illustrated the power of a genome-wide CRISPR-Cas9 screen to systematically identify novel genes involved in APAP-induced hepatocyte toxicity and to provide potential new targets to develop novel therapeutic modalities. Combined with functional validations, this screening technique offers a robust and dynamic way to identify candidate genes for a variety of disease models. In this study we demonstrate that *LZTR1* and *PGM*5 knockout and knockdown are protective against APAP–induced hepatotoxicity.

The gene *NAMPT* is protective against APAP-induced ALI *in vivo*, although not identified directly by the sgRNA screen, we show knockdown increases susceptibility to APAP-induced hepatotoxicity. NAMPT has a known role in NAD salvage that warrants further study to identify if its protective effect is resultant of increased NAD supporting glutathione production and CYP function, or if it is protective by a novel mechanism.

These genes represent novel diagnostic and therapeutic targets for improving the care of acetaminophen overdose. Gene expression could be used to determine susceptibility to APAP-hepatotoxicity as well diagnose and predict disease severity and outcome. Expression and function-associated variants in these genes could be used in risk-assessment genotyping panels. Furthermore, these genes represent novel biomarkers for personalized therapeutics. In silico analysis of candidate genes identified a number of the candidate genes that are targets for existing drugs. These existing drugs could be quickly re-purposed to treat and prevent APAP-induced ALF. Further studies are needed to better understand the functional role of the genes and pathways highlighted in this study.

## Methods

### GeCKOv2 sgRNA library

The genome-wide CRISPR-Cas9 gene knockdown screen was accomplished using HuH7 human hepatoma cells and the GeCKOv2 gene knockout library^23,63–65^. The human GeCKOv2 sgRNA libraries A and B contained 122,411 targeting sgRNA and 1,000 non-targeting control sgRNA, of which 119,461 were unique sgRNAs (117,481 targeting sgRNAs). Libraries A and B were amplified in Endura competent cells (Lucigen cat. 60242-1, Middleton, WI) and isolated using the Purelink HiPure plasmid midi prep kit (Invitrogen k210005, Carlsbad, CA) as previously described^23,64^.

### Cell Culture

HEK293FT cells (Thermo Fisher cat. R70007, Waltham, MA) were maintained in high-glucose DMEM (Thermo Fisher cat. 11965118) supplemented with 100 U/ml penicillin and streptomycin (Thermo Fisher cat. 15140122), non-essential amino acids (Thermo Fisher cat. 11140050), 2 mM L-glutamine (Thermo Fisher cat. 25030081), 1 mM sodium pyruvate (Thermo Fisher cat. 11360070), and 10% fetal bovine serum (Atlanta Biologicals cat. S11150, Atlanta, GA). Cells were detached with trypsin-EDTA (Thermo Fisher cat. 25200056).

HuH7 was obtained from the Japanese Collection of Research Bioresources Cell Bank^66^. The HuH7 human hepatocellular carcinoma cell line (JCRB cat. 0403, Osaka, Japan) was chosen as a model for APAP toxicity studies because it is more robust than primary hepatocytes, allowing efficient lentiviral transduction, transfection, and genome editing with CRISPR/Cas9^62,67–70^.

Cells were maintained in DMEM (Thermo Fisher cat. 111885092) supplemented with 100 U/ml penicillin and streptomycin (Thermo Fisher cat. 15140122), non-essential amino acids (Thermo Fisher cat. 11140050), and 10% fetal bovine serum (Atlanta Biologicals cat. S11150) as previously described, with the addition of 2 mM L-glutamine (Thermo Fisher cat. 25030081) and 1 mM sodium pyruvate (Thermo Fisher)^71^. Cells were detached with trypsin-EDTA (Thermo Fisher cat. 25200056). All incubations were performed at 37 °C and 5% CO_2_.

### Lentivirus Production and Purification to Produce Lentivirus

T-150 TPP flasks (18 T-150 flasks for the library, MidSci cat. TP0151, Valley Park, MO) of HEK293T cells were seeded at ~40% confluence the day before transfection in DMEM. One hour prior to transfection, media was removed and 18 mL of pre-warmed reduced serum OptiMEM media (Thermo Fisher cat. 31985070) was added to each flask. Transfection was performed using Lipofectamine 2000 (Thermo Fisher cat. 11668019) and Plus reagent (Thermo Fisher cat. 11514015). For each flask, 200 μl of Plus reagent was diluted in 3 ml OptiMEM with 20 μg of lentiCRISPR plasmid library, 10 μg of pVSVg, and 15 μg of psPAX2. 100 μl of Lipofectamine 2000 was diluted in 3 ml OptiMEM and, after 5 min, it was added to the mixture of DNA and Plus reagent. The complete mixture was incubated for 20 min before being added to cells. After 6 h, the media was changed to 24 ml D10 supplemented with 1% BSA (Sigma cat. A8412-100ML, St. Louis, MO). After 84 h, the media was removed and centrifuged at 3,000 × g at 4 °C for 5 min to pellet cell debris. The supernatant was filtered through a 0.45 um low protein binding membrane (EMD Millipore Steriflip cat. SE1MO03MO0 or stericup cat. SCHVU05RE, Billerica, MA). To achieve concentration of the GeCKO v2 pooled library, the virus was ultracentrifuged (Beckman-Coulter, Brea, CA) at 84,000 × g for 1 h at 4 °C and then re-suspended overnight at 4 °C in D10 supplemented with 1% BSA. Aliquots were stored at −80 °C. Lentiviruses were titrated by qRT-PCR (Clontech Lenti-X™ qRT-PCR Titration Kit cat. 631235, Mountain View, CA).

### APAP Kill Curve

The APAP concentration used for the screen was determined by measuring cell proliferation of HuH7 stably transduced with Cas9 and Guide-Puro (empty vector) in the presence of 0–20 mM APAP (Sigma cat. A7085. St. Louis, MO) daily for 7 days. HuH7 were seeded at 20,000 cells/96-well (MidSci cat. TP92696, Valley Park, MO) prior to APAP treatment. Titration of APAP concentrations ranging from 5 mM–20 mM was accomplished by measuring cell count at 24 hour intervals for seven days by trypan blue counting (Sigma cat. T8154-100ML, St. Louis, MO). Percent of cell death was determined as an average of cell count divided by untreated cell count (N = 3). For the screen, 15 mM APAP was chosen because there were 95% fewer cells at 3 days selection than mock and 99% fewer cells at day 4 than mock, based on the strategy of Wang *et al*.^27^.

### Cell Transduction Using the GeCKOv2 Library

HuH7 cells were detached using 0.25% Trypsin-EDTA (Thermo Fisher cat. 25200056) and seeded the day prior to transduction at 6E6 cells per T-150 TPP flask (MidSci cat. TP0151, Valley Park, MO). The flasks were then transduced for 48 h in culture media + 8 µg/ml polybrene (Thermo Fisher cat. 107689-10 G) + Cas9 lentivirus at an MOI  <0.1. HuH7 underwent monoclonal selection by 1 ug/ml blasticidin (Thermo Fisher cat. A1113903) before Cas9 expression was confirmed by western blot. HuH7-Cas9 was transfected with the GeCKOv2 packaged lentiviral library as described above at 0.5 MOI. The pooled, transduced cells were selected with 1.5 µg/ml puromycin (Invitrogen cat. Ant-pr-1) for 3 days alongside cells transduced with the empty vector lentiGuidePuro, positive fluorescent control PLJM1-EGFP. PLJM1-EGFP fluorescence was verified 48 h post-transduction.

### APAP Screen and Sample Collection

After 8 days of transduction a T0 sample was collected (N = 2) and the remaining library-transduced cells were treated with 15 mM APAP for 30 minutes up to 4 days (2 biological replicates for T0, 24 hour, and 4 day samples). Samples that underwent 4 days of APAP treatment were outgrown for 21 days prior to collection. Genomic DNA was isolated from samples of a minimum of 2E7 cells using the Blood and Cell Culture Midi Kit (Qiagen cat. 13343, Valencia, CA), resulting in a minimum of 136 µg DNA per sample. DNA was quantified using the Qubit high-sensitivity DNA quantification assay (Thermo Fisher cat. Q32851) and Take3 microspot plate reader (BioTek Epoch, Winooski, VT).

### Sequencing

3.33 µg of the isolated genomic DNA was used to amplify the bar-coded amplicons in 39 Herculase II DNA polymerase (Agilent cat. 600679, Santa Clara, CA) reactions per sample (primers described in Supplementary Data 13). 5 µl amplicon or 1 µl diluted plasmid library was used as template in 13 50 µl Herculase II DNA polymerase reactions per sample to attach pooled variable-length spacers and Illumina indexes (primers described in Supplementary Data 13). 24 cycles were used to amplify DNA in the first and second PCR, respectively. The amplicon fragments after PCR 2 have the following sequence (354–362 bp library with variable 20 bp sgRNA sequence in the middle) (SF1). DNA was pooled by sample and purified using the Nucleospin Gel and PCR Clean-up kit (Clontech cat. 740609.250, Mountain View, CA). DNA was quantified using a Qubit high-sensitivity DNA quantification assay (Thermo Fisher cat. Q32851) and Take3 microspot plate reader (BioTek). DNA quality was analyzed by Experion CHIP assay (BioRad cat. 7007-163, Hercules, CA). Clusters were generated on the flow cell using the HiSeq Rapid Duo CBot Sample Loading Kit (Illumina CT- cat. 403-2001, San Diego, CA). A single-read rapid run of 75 cycles was performed on a HiSeq. 1500 (Illumina cat. GD-402-4002) using the HiSeq Rapid SBS kit (Illumina cat. FC-402-4022) with 10% PhiX.

### GeCKOv2 screen deconvolution and statistical analysis

The sequence reads were demultiplexed and converted to fastQ with BCL2FastQ v2.17 (Illumina) and trimmed in cutadapt 1.7.1 (with Python 2.7.6) to only the 20 bp sgRNA^72^. Trimmed reads were aligned to the index in Bowtie2 v2.1 with a 1 bp mismatch allowance^73^. Read counts were normalized to the median with T0 as control and analyzed using sgRNA and gene-level RRA (Robust Rank Aggregation) in MaGeCK v0.5.6. In comparisons between 2 time points the biological replicates were handled as independent replicates and in the pooled T0 vs. 30 min–24 h and 30 min-end the replicates were combined. Gene-level analysis was validated using Maximum Likelihood Estimate (MLE) in MaGeCK v0.5.6. Genes with fewer than 3 sgRNA were removed from the gene-level analysis but were included in the Gene Set Enrichment Analysis (GSEA) pathway analysis implemented in MaGeCK v0.5.6^32,74^. Box plot, scatter plots and heat map were generated in R. Venn-diagrams were generated using http://bioinformatics.psb.ugent.be/webtools/Venn/.

### Pathway analysis

Analysis of pathway-level effects of APAP treatment in the 24 h and 4d samples individually vs. T0 was accomplished using GSEA in Mageck v0.5.6 using the MsigDB “KEGG gene sets” and “all GO gene sets”. Ingenuity Pathway Analysis of 24 h vs. T0 (genes with p < 0.05) and 4d vs. T0 (genes with p < 0.05) was also used to predict pathway-level effects of APAP treatment.

### Statistical analysis of GEO datasets

#### Human APAP analysis

We then analyzed samples from 2 publicly available human datasets of acetaminophen overdose from the Gene Expression Omnibus, GSE74000 and GSE70784^9,75^. Gene candidates identified using the genome-wide CRISPR-Cas9 screen were cross-referenced with gens that were significantly correlated with APAP overdose from 2 human microarray datasets identified in the Gene Expression Omnibus (https://www.ncbi.nlm.nih.gov/geo/).

Of the available gene expression datasets assessing the effect of APAP, these were selected because they address hepatotoxicity at a range of stages. These datasets were analyzed in GEO2R using the microarray data normalized and deposited by the original authors. GSE70784 contains gene-expression data from blood in patients receiving a daily dose of APAP or placebo. These data compare patients at a higher risk of injury (responders) to non-responders and placebo after 1 day and 8 days of dosing. Genes with differential expression in blood, especially early after dosing, are ideal diagnostic biomarkers. GSE7400 contains gene expression data from liver biopsies from healthy patients and patients APAP-induced-ALF. These data address differential gene expression in end-stage disease, and better inform the biological mechanisms active in APAP-induced ALF.

In GEO2R, microarray data from 12 APAP responder blood samples were compared to 32 non-responders and 10 placebo controls on 1 day and 8 days of APAP treatment (GSE70784). Subjects were treated with 4 g APAP or placebo for 7 days and were followed for 14 days. Responders were classified as patients with ALT (alanine aminotransferase). >2 times the upper limit of normal during days 4–9 after the start of APAP dosing. Background correction and normalization was completed by the depositing authors. Data was log_2_ transformed prior to analysis and the unadjusted p-values were used for comparison with the CRISPR screen.

Microarray data from 3 APAP-induced ALF liver samples were compared to 2 healthy liver samples were obtained from the GEO dataset GSE74000 and compared using GEO2R. Background correction, median polish summarization, and quantile normalization were completed by the depositing authors. Data was log_2_ transformed prior to analysis and the FDR-adjusted p-values were used for comparison with the CRISPR screen. Heat maps were generated in R. Box plots were generated in GEO2R.

#### Mouse APAP analysis

RNA-seq data from mice previously published by our lab (GSE110787) evaluating the effect of APAP overdose on RNA expression changes in the liver was 7 male 11 week old C57BL/6 mice, 4 saline treated control mice and 3 mice 24 h after 200 mg/kg APAP (Sigma cat. A7085, St. Louis, MO) exposure via intraperitoneal injection, underwent RNA-sequencing on an Illumina HiSeq 1500^35^. RNA was isolated from liver using the MirVana miRNA isolation kit (Thermo Fisher cat. AM1561, Waltham, MA).

Samples were prepared using the TruSeq Stranded Total RNA Sample Preparation Kit (Illumina cat. RS-122-2201, San Diego, CA) and clusters were generated using the TruSeq Paired-End Cluster Kit v3-cBot-HS (Illumina cat. PE-401-3001, San Diego, CA). Paired –end sequencing (2 × 101 cycles) was completed using the TruSeq SBS kit v3-HS (Illumina cat. FC-401-3001, San Diego, CA). The raw base calling (.bcl) files were converted to demultiplexed compressed FASTQ files using Illumina’s bcl2fastq v2.17 software. TopHat 2.0.9 was used to map RNA-seq reads against the mouse reference genome (mm10) using default parameters^76,77^. Transcript assembly and abundance estimation and comparing expression were conducted with Cufflinks v2.2.1 and reported in Fragments Per Kilobase of exon per Million fragments mapped (FPKM). Cuffdiff, a part of the Cufflinks package, was used to calculate statistical significance changes of gene expression between treated and untreated mice. Box plot and heat maps were generated in R.

This RNA-seq study of APAP-induced ALI identified genes which were differentially expressed in a genetically and drug dosage controlled environment after liver injury has occurred, but prior to ALF. These data better illustrate the changes in gene expression due to the drug overdose absent of the variation that is unavoidable in human studies.

### Functional validations in primary mouse hepatocytes and analysis

Cryopreserved hepatocytes (Lonza cat. MBCP01, Allendale, NJ) from 8-week old male C57/Bl6 mice were thawed in thawing media (Lonza. Cat. MCRT50) and immediately seeded at a density of 15,000 cells/96-well and 250,000 cells/12-well in Williams E media with thawing and plating supplement (Thermo cat. A1217601, cat. CM3000, respectively). After 4 h the cells were transfected using the standard Polyplus INTERFERin protocol for 4 h (VWR cat. 89129-930, Radnor PA) and 25 nM TYE-563 fluorescent control (IDT cat. 51-01-20-19) or SmartPool scrambled siRNA or (*Nampt*, *Lztr1*, *Naaa*) siRNA (Dharmacon, Lafayette, CO) or 50–100 nM SmartPool siRNA (*Pgm5*, Dharmacon) in Williams E media with thawing and plating supplement (serum-free, Thermo cat. A1217601, cat. CM4000, respectively). 20 hours after transfection TYE-563 positive fluorescent controls were imaged and cells were treated with +/−7.5 mM APAP for 3 h beginning 22 h post-transfection. Cell viability was measured by ATP luminescence read at 0.25 seconds with n = 6 and the high and low values removed for a final n = 4 (Promega CellTiter-Glo cat. G7571, Madison, WI) using a TriStar LB 941 Multimode Microplate Reader (Berthold Technologies, Bad Wildbad, Germany). For each siRNA transfection, APAP-treated wells were normalized to untreated wells. Statistical significance was determined by a 2 sample 2-tailed Student’s t-test assuming equal variance (p < 0.05). Gene expression was validated by sqPCR (primers are described in Supplementary Data 13).

### Western blotting

HuH7 cell lysates were collected on ice in RIPA buffer and isolated by centrifugation at ~16,000 × g for 10 minutes @ 4 °C. Protein was quantified by Pierce BCA (Thermo Fisher cat. 23225, Waltham, MA). 30 µg protein (western) was boiled with sample buffer prior to loading on a polyacrylamide gel. Cas9 antibody diluted 1:2,000 in TBS-T + 3% milk (EMD Millipore cat. MAC133, Billerica, MA), GAPDH antibody diluted 1:2,000 in TBS-T + 5% milk (Santa Cruz cat. sc-25778, Santa Cruz, CA). Goat anti-rabbit HRP antibody 1:10,000 in TBS-T + 5% milk (Vector Biolabs cat. PI-1000, Malvern, PA) and horse anti-mouse HRP antibody in TBS-T + 5% milk (vector Biolabs cat. P1-2000, Malvern, PA) were used to visualize GAPDH and Cas9. Band size was visualized using the Precision Plus Protein Dual Color Standard (Bio-Rad cat. 161-0374).

### sqPCR

RNA was isolated using the MirVana miRNA isolation kit (Thermo Fisher cat. AM1561, Waltham, MA) and quantified using the Epoch Take3 (BioTek, Winooski, VT). cDNA was amplified from 500 ng mRNA by SuperScript IV (Thermo Fisher cat. 18091200, Waltham, MA). 2 µl CDNA was used as sqPCR template using Platinum Taq polymerase (Thermo Fisher cat. 10966-026, Waltham, MA) (primers are described in Supplementary Data 13). A 2.5% agarose gel was run @100 V to visualize knockdown of mouse *Lztr1*, *Nampt*, and *Pgm5* with *ActB* used as a loading control.

### Drug-Gene interaction analysis

Genes in the top 10 of a CRISPR-Cas9 knockout screen list and overlapping a gene expression dataset (p < 0.05), in a CRISPR-Cas9 knockout screen list (p < 0.05) and involved in NAD metabolism, or in a CRISPR-Cas9 knockout screen list (p < 0.05) and containing or nearest neighbor to APAP-associated SNPs (Supplementary Data 13 and Tables 5–6) were compared against the Drug Gene Interaction Database (http://www.dgidb.org/) to assess known drug interactions and potential re-purposing of existing drugs^78^.

### Plasmids

The lenti Guide_puro backbone, lenti Cas9_blast, and the Human GeCKOv2 CRISPR knockout pooled library were obtained from Addgene (pooled library #1000000048, #1000000049, plasmid #52962, 52963 originally from Feng Zhang’s lab, respectively)^64^. psPAX2 was a gift from Didier Trono (Addgene plasmid # 12260; http://n2t.net/addgene:12260; RRID:Addgene_12260). pCMV-VSV-G was a gift from Bob Weinberg (Addgene plasmid # 8454; http://n2t.net/addgene:8454; RRID:Addgene_8454)^79^. pLJM1-EGFP was a gift from David Sabatini (Addgene plasmid # 19319; http://n2t.net/addgene:19319; RRID:Addgene_19319)^80^.

### Source of Data

All research has been approved by the University of Missouri Kansas City Institutional Biosafety Committee. The studies using APAP overdose and acute liver failure patient microarray data are not classified as human subjects research because the data are previously collected and de-identified.

## Supplementary information

Supplementary Information

Supplementary Datasets 1–13

## Data Availability

Amplicon sequence data from the CRISPR-Cas9 screen has been submitted to the Gene Expression Omnibus (https://www.ncbi.nlm.nih.gov/geo/, accession # GSE112463, accession # GSE112463).

## References

[1] Chun LJ, Tong MJ, Busuttil RW, Hiatt JR (2009). Acetaminophen hepatotoxicity and acute liver failure. Journal of clinical gastroenterology.

[2] Lee WM (2004). Acetaminophen and the US Acute Liver Failure Study Group: lowering the risks of hepatic failure. Hepatology (Baltimore, Md.).

[3] Zhao P (2013). Causes and outcomes of acute liver failure in China. Plos one.

[4] Farrell, S. E. *et al*. Acetaminophen Toxicity. *Medscape* (2014).

[5] Lancaster EM, Hiatt JR, Zarrinpar A (2015). Acetaminophen hepatotoxicity: an updated review. Archives of toxicology.

[6] Jiang J (2015). Increased mitochondrial ROS formation by acetaminophen in human hepatic cells is associated with gene expression changes suggesting disruption of the mitochondrial electron transport chain. Toxicology letters.

[7] Sjogren AK (2014). Critical differences in toxicity mechanisms in induced pluripotent stem cell-derived hepatocytes, hepatic cell lines and primary hepatocytes. Archives of toxicology.

[8] Moyer, A. M. *et al*. Acetaminophen-NAPQI hepatotoxicity: a cell line model system genome-wide association study. *Toxicol Sci* 120, 33–41, 10.1093/toxsci/kfq375. 10.1093/toxsci/kfq375 PMC3044203 21177773

[9] Bushel, P. R., Fannin, R. D., Gerrish, K., Watkins, P. B. & Paules, R. S. Blood gene expression profiling of an early acetaminophen response. *The pharmacogenomics journal*, 10.1038/tpj.2016.8 (2016). 10.1038/tpj.2016.8 PMC5782799 26927286

[10] Ruepp SU, Tonge RP, Shaw J, Wallis N, Pognan F (2002). Genomics and proteomics analysis of acetaminophen toxicity in mouse liver. Toxicol Sci.

[11] Reilly TP (2001). Expression profiling of acetaminophen liver toxicity in mice using microarray technology. Biochemical and biophysical research communications.

[12] Fukushima T, Hamada Y, Yamada H, Horii I (2007). Changes of micro-RNA expression in rat liver treated by acetaminophen or carbon tetrachloride–regulating role of micro-RNA for RNA expression. The Journal of toxicological sciences.

[13] Fannin RD (2010). Acetaminophen dosing of humans results in blood transcriptome and metabolome changes consistent with impaired oxidative phosphorylation. Hepatology (Baltimore, Md.).

[14] Paddison PJ (2004). A resource for large-scale RNA-interference-based screens in mammals. Nature.

[15] Deans RM (2016). Parallel shRNA and CRISPR-Cas9 screens enable antiviral drug target identification. Nature chemical biology.

[16] Morgens DW, Deans RM, Li A, Bassik MC (2016). Systematic comparison of CRISPR/Cas9 and RNAi screens for essential genes. Nature biotechnology.

[17] Bibikova M (2001). Stimulation of homologous recombination through targeted cleavage by chimeric nucleases. Molecular and cellular biology.

[18] Urnov FD (2005). Highly efficient endogenous human gene correction using designed zinc-finger nucleases. Nature.

[19] Boch J (2009). Breaking the code of DNA binding specificity of TAL-type III effectors. Science (New York, N.Y.).

[20] Christian M (2010). Targeting DNA double-strand breaks with TAL effector nucleases. Genetics.

[21] Riordan SM, Heruth DP, Zhang LQ, Ye SQ (2015). Application of CRISPR/Cas9 for biomedical discoveries. Cell & bioscience.

[22] Xue HY (2016). CRISPR-Cas9 for medical genetic screens: applications and future perspectives. Journal of medical genetics.

[23] Shalem O (2014). Genome-scale CRISPR-Cas9 knockout screening in human cells. Science (New York, N.Y.).

[24] Gilbert LA (2014). Genome-Scale CRISPR-Mediated Control of Gene Repression and Activation. Cell.

[25] Konermann S (2015). Genome-scale transcriptional activation by an engineered CRISPR-Cas9 complex. Nature.

[26] Chen S (2015). Genome-wide CRISPR screen in a mouse model of tumor growth and metastasis. Cell.

[27] Wang T, Wei JJ, Sabatini DM, Lander ES (2014). Genetic screens in human cells using the CRISPR-Cas9 system. Science (New York, N.Y.).

[28] Du D (2017). Genetic interaction mapping in mammalian cells using CRISPR interference. Nature methods.

[29] Wang T (2015). Identification and characterization of essential genes in the human genome. Science (New York, N.Y.).

[30] Banerjee S (2017). Trifluoperazine inhibits acetaminophen-induced hepatotoxicity and hepatic reactive nitrogen formation in mice and in freshly isolated hepatocytes. Toxicology reports.

[31] Holownia A, Menez JF, Braszko JJ (1998). The role of calcium in paracetamol (acetaminophen) cytotoxicity in PC12 cells transfected with CYP4502E1. Inflammopharmacology.

[32] Li W (2014). MAGeCK enables robust identification of essential genes from genome-scale CRISPR/Cas9 knockout screens. Genome biology.

[33] Li W (2015). Quality control, modeling, and visualization of CRISPR screens with MAGeCK-VISPR. Genome biology.

[34] Nikiforov A, Kulikova V, Ziegler M (2015). The human NAD metabolome: Functions, metabolism and compartmentalization. Critical reviews in biochemistry and molecular biology.

[35] Zhang L (2018). Novel Protective Role of Nicotinamide Phosphoribosyltransferase in Acetaminophen-induced Acute Liver Injury in Mice. Am J Pathol.

[36] Heruth, D. P. *et al*. Genetic Association of Single Nucleotide Polymorphisms with Acetaminophen-Induced Hepatotoxicity. *The Journal of pharmacology and experimental therapeutics* **367**, 95–100, 10.1124/jpet.118.248583 (2018). 10.1124/jpet.118.248583 30076262

[37] Zhang J, Huang W, Chua SS, Wei P, Moore DD (2002). Modulation of acetaminophen-induced hepatotoxicity by the xenobiotic receptor CAR. Science (New York, N.Y.).

[38] Lu Z (2011). SIRT3-dependent deacetylation exacerbates acetaminophen hepatotoxicity. EMBO reports.

[39] Henderson CJ (2000). Increased resistance to acetaminophen hepatotoxicity in mice lacking glutathione S-transferase Pi. Proceedings of the National Academy of Sciences of the United States of America.

[40] Rada, P. *et al*. SIRT1 Controls Acetaminophen Hepatotoxicity by Modulating Inflammation and Oxidative Stress. *Antioxidants & redox signaling*, 10.1089/ars.2017.7373 (2017). 10.1089/ars.2017.7373 PMC9545809 29084443

[41] Lorincz T, Jemnitz K, Kardon T, Mandl J, Szarka A (2015). Ferroptosis is Involved in Acetaminophen Induced Cell Death. Pathology oncology research: POR.

[42] Chan K, Han XD, Kan YW (2001). An important function of Nrf2 in combating oxidative stress: detoxification of acetaminophen. Proceedings of the National Academy of Sciences of the United States of America.

[43] Jiang L (2017). Blockade of Notch signaling promotes acetaminophen-induced liver injury. Immunologic research.

[44] Woolbright BL, Jaeschke H (2017). Role of the inflammasome in acetaminophen-induced liver injury and acute liver failure. Journal of hepatology.

[45] Nacak TG, Leptien K, Fellner D, Augustin HG, Kroll J (2006). The BTB-kelch protein LZTR-1 is a novel Golgi protein that is degraded upon induction of apoptosis. The Journal of biological chemistry.

[46] Yamamoto GL (2015). Rare variants in SOS2 and LZTR1 are associated with Noonan syndrome. Journal of medical genetics.

[47] Piotrowski A (2014). Germline loss-of-function mutations in LZTR1 predispose to an inherited disorder of multiple schwannomas. Nature genetics.

[48] Fu F (2017). Clinical application of SNP array analysis in fetuses with ventricular septal defects and normal karyotypes. Arch Gynecol Obstet.

[49] Bauer, L. *et al*. A novel pretherapeutic gene expression based risk score for treatment guidance in gastric cancer. *Ann Oncol*, 10.1093/annonc/mdx685 (2017). 10.1093/annonc/mdx685 29069277

[50] Garten A (2015). Physiological and pathophysiological roles of NAMPT and NAD metabolism. Nature reviews. Endocrinology.

[51] Zhang, M. & Ying, W. NAD+ deficiency is a common central pathological factor of a number of diseases and aging: Mechanisms and therapeutic implications. *Antioxidants & redox signaling*, 10.1089/ars.2017.7445 (2018). 10.1089/ars.2017.7445 29295624

[52] Blakemore AI (2009). A rare variant in the visfatin gene (NAMPT/PBEF1) is associated with protection from obesity. Obesity (Silver Spring, Md.).

[53] Saddi-Rosa P (2013). Association of circulating levels of nicotinamide phosphoribosyltransferase (NAMPT/Visfatin) and of a frequent polymorphism in the promoter of the NAMPT gene with coronary artery disease in diabetic and non-diabetic subjects. Cardiovascular diabetology.

[54] Aller R (2009). Influence of visfatin on histopathological changes of non-alcoholic fatty liver disease. Digestive diseases and sciences.

[55] Revollo JR (2007). Nampt/PBEF/Visfatin regulates insulin secretion in beta cells as a systemic NAD biosynthetic enzyme. Cell Metab.

[56] van der Veer E (2007). Extension of human cell lifespan by nicotinamide phosphoribosyltransferase. The Journal of biological chemistry.

[57] Hasmann M, Schemainda I (2003). FK866, a highly specific noncompetitive inhibitor of nicotinamide phosphoribosyltransferase, represents a novel mechanism for induction of tumor cell apoptosis. Cancer Res.

[58] Jia SH (2004). Pre-B cell colony-enhancing factor inhibits neutrophil apoptosis in experimental inflammation and clinical sepsis. The Journal of clinical investigation.

[59] Wakayama Y (2000). Aciculin and its relation to dystrophin: immunocytochemical studies in human normal and Duchenne dystrophy quadriceps muscles. Acta neuropathologica.

[60] Uzozie AC (2017). Targeted Proteomics for Multiplexed Verification of Markers of Colorectal Tumorigenesis. Molecular & cellular proteomics: MCP.

[61] Lin J (2012). Comparative analysis of phase I and II enzyme activities in 5 hepatic cell lines identifies Huh-7 and HCC-T cells with the highest potential to study drug metabolism. Archives of toxicology.

[62] Choi S, Sainz B, Corcoran P, Uprichard S, Jeong H (2009). Characterization of increased drug metabolism activity in dimethyl sulfoxide (DMSO)-treated Huh7 hepatoma cells. Xenobiotica; the fate of foreign compounds in biological systems.

[63] Shalem O, Sanjana NE, Zhang F (2015). High-throughput functional genomics using CRISPR-Cas9. Nat Rev Genet.

[64] Sanjana NE, Shalem O, Zhang F (2014). Improved vectors and genome-wide libraries for CRISPR screening. Nature methods.

[65] Joung J (2017). Genome-scale CRISPR-Cas9 knockout and transcriptional activation screening. Nature protocols.

[66] Nakabayashi H, Taketa K, Miyano K, Yamane T, Sato J (1982). Growth of human hepatoma cells lines with differentiated functions in chemically defined medium. Cancer research.

[67] Scheiermann P (2015). Application of IL-36 receptor antagonist weakens CCL20 expression and impairs recovery in the late phase of murine acetaminophen-induced liver injury. Scientific reports.

[68] Mobasher MA (2014). Essential role of protein-tyrosine phosphatase 1B in the modulation of insulin signaling by acetaminophen in hepatocytes. The Journal of biological chemistry.

[69] Macanas-Pirard P (2005). Glycogen synthase kinase-3 mediates acetaminophen-induced apoptosis in human hepatoma cells. The Journal of pharmacology and experimental therapeutics.

[70] Olsavsky KM (2007). Gene Expression Profiling and Differentiation Assessment in Primary Human Hepatocyte Cultures, Established Hepatoma Cell Lines, and Human Liver Tissues. Toxicology and applied pharmacology.

[71] Blight KJ, McKeating JA, Rice CM (2002). Highly permissive cell lines for subgenomic and genomic hepatitis C virus RNA replication. Journal of virology.

[72] Martin M (2011). Cutadapt removes adapter sequences from high-throughput sequencing reads. 2011.

[73] Langmead B, Salzberg SL (2012). Fast gapped-read alignment with Bowtie 2. Nature methods.

[74] Subramanian A (2005). Gene set enrichment analysis: a knowledge-based approach for interpreting genome-wide expression profiles. Proceedings of the National Academy of Sciences of the United States of America.

[75] Rodrigues RM (2016). Toxicogenomics-based prediction of acetaminophen-induced liver injury using human hepatic cell systems. Toxicology letters.

[76] Trapnell C, Pachter L, Salzberg SL (2009). TopHat: discovering splice junctions with RNA-Seq. Bioinformatics (Oxford, England).

[77] Trapnell C (2012). Differential gene and transcript expression analysis of RNA-seq experiments with TopHat and Cufflinks. Nature protocols.

[78] Cotto, K. C. *et al*. DGIdb 3.0: a redesign and expansion of the drug-gene interaction database. *Nucleic acids research*, 10.1093/nar/gkx1143 (2017). 10.1093/nar/gkx1143 PMC5888642 29156001

[79] Stewart SA (2003). Lentivirus-delivered stable gene silencing by RNAi in primary cells. RNA (New York, N.Y.).

[80] Sancak, Y. *et al*. The Rag GTPases bind raptor and mediate amino acid signaling to mTORC1. *Science (New York*, *N*.*Y*.*)* 320, 1496–1501, 10.1126/science.1157535 (2008). 10.1126/science.1157535 PMC2475333 18497260

