# Supplementary material for: Identification of Novel Regulatory Genes in APAP Induced Hepatocyte Toxicity by a Genome-Wide CRISPR-Cas9 Screen
Source: Sci Rep. 2019 Feb 4;9:1396. doi: 10.1038/s41598-018-37940-6 (PMC6362041; doi:10.1038/s41598-018-37940-6)
Supplement: Supplementary file 1 — Supplementary Information [file 41598_2018_37940_MOESM1_ESM.pdf]

Supplementary Information

Description of supplementary data files, supplementary figures, and supplementary tables that accompany this manuscript.

Identification of Novel Regulatory Genes in APAP Induced Hepatocyte Toxicity by a Genome-Wide CRISPR-Cas9 Screen

Katherine Shortt[1,3,5,*], Daniel P. Heruth[1,#], NiNi Zhang[1,2,7], Weibin Wu[1,3], Shipra Singh[1,3], Ding-You Li[2], Li Qin Zhang[1,4,#], Gerald J. Wyckoff[6], Lei S. Qi[8], Craig A. Friesen[2], Shui Qing Ye[1,3,5,†]

[1]Division of Experimental and Translational Genetics and Division of Gastroenterology, Hepatology, and Nutrition[2], Children's Mercy Kansas City, Kansas City, MO, USA, University of Missouri Kansas City School of Medicine, Kansas City, MO, USA

[3]Department of Biomedical and Health Informatics and Department of Biomedical Sciences[4], University of Missouri Kansas City School of Medicine, Kansas City, MO, USA

[5]Division of Cell Biology and Biophysics and Division of Molecular Biology & Biochemistry[6], University of Missouri Kansas City School of Biological Sciences, Kansas City, MO, USA

[7]Department of Pediatrics, Tangdu Hospital, The Fourth Military Medical University, Xian, China

[8]Department of Bioengineering, Department of Chemical and Systems Biology, ChEM-H, Stanford University, Stanford, CA 94305, USA.

*Current Address: Precision Genomics, Intermountain Healthcare St. George, UT, 84790, USA

†Deceased.

#Address correspondence to:

Daniel P. Heruth, PhD
Department of Pediatrics
Children's Mercy Kansas City
2401 Gillham Road
Kansas City, MO, USA, 64108
E-mail: dpheruth@cmh.edu
Tel. No. (816)983-6502
Fax: (816)983-6501

Li Qin Zhang, MD
Department of Biomedical Sciences
University of Missouri Kansas City School of Medicine
2411 Holmes Street

Kansas City, MO, USA 64108
E-mail: zhanglq@umkc.edu
Tel. No. (816)235-5284
Fax: (816)235-1074

Description of Additional Supplementary Data Files

File Name: Supplementary Data 1
Description: Gene knockouts ranked by RRA analysis of the CRISPR-Cas9 screen 30min-4d APAP treatment vs. T0.

File Name: Supplementary Data 2
Description: Gene knockouts ranked by RRA analysis of the CRISPR-Cas9 screen 30min-24h APAP treatment vs. T0.

File Name: Supplementary Data 3
Description: Gene knockouts ranked by RRA analysis of the CRISPR-Cas9 screen 30min APAP treatment vs. T0.

File Name: Supplementary Data 4
Description: Gene knockouts ranked by RRA analysis of the CRISPR-Cas9 screen 3h APAP treatment vs. T0.

File Name: Supplementary Data 5
Description: Gene knockouts ranked by RRA analysis of the CRISPR-Cas9 screen 6h APAP treatment vs. T0.

File Name: Supplementary Data 6
Description: Gene knockouts ranked by RRA analysis of the CRISPR-Cas9 screen 12h APAP treatment vs. T0.

File Name: Supplementary Data 7
Description: Gene knockouts ranked by RRA analysis of the CRISPR-Cas9 screen 24h APAP treatment vs. T0.

File Name: Supplementary Data 8
Description: Gene knockouts ranked by RRA analysis of the CRISPR-Cas9 screen 4d APAP treatment vs. T0.

File Name: Supplementary Data 9
Description: Genes ranked by Maximum Likelihood Estimate comparing all APAP samples to T0.

File Name: Supplementary Data 10
Description: Genes differentially expressed with and without APAP treatment from RNA-sequenced mice ($p<0.05$).

File Name: Supplementary Data 11
Description: Summary information about the top genes considered for further studies. Genes had to be ranked in the top 10 of a CRISPR screen gene list and also be significantly differentially expressed in another dataset (all $p<0.05$).

File Name: Supplementary Data 12
Description: All Drug-Gene interactions resultant from analysis of candidate genes by the Drug Gene Interaction Database (www.dgidb.org)

File Name: Supplementary Data 13
Description: Primers used for sequencing, cloning, and sqPCR.

Supplementary Information

Supplementary figures and tables that accompany this manuscript

Supplementary Figures

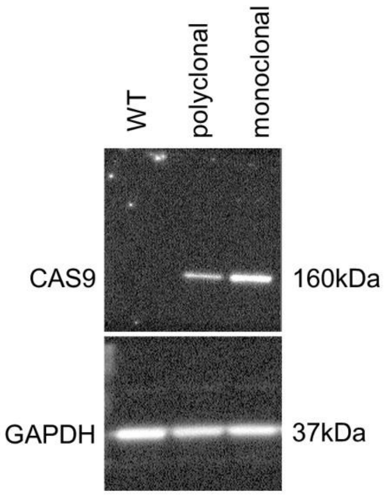

Supplementary Figure 1 | Full-length western blot of Cas9 and GAPDH shown in Figure 1. Expression levels of Cas9 in untransduced, polyclonal, and Monoclonal HuH7-Cas9 cell line.

Illumina F(P5 and seq)
Binding site- F indexing primer
Binding site- R indexing primer
Illumina R(seq)
Illumina P7

5'-
AATGATACGGCGACCACCGAGATCTACACTCTTTCCCTAC
ACGACGCTCTTCCGATCT (1-9bp heterogeneity spacer)
TCTTGTGGAAAGGACGAAACACCGNNNNNNNNNNNNNN
NNNNNNGTTTTAGAGCTAGAAATAGCAAGTTAAAATAAGG
CTAGTCCGTTATCAACTTGAAAAAGTGGCACCGAGTCGG
TGCTTTTTTAAGCTTGGCGTAACTAGATCTTGAGACAAAT
GGCAGTATTCATCCACAATTTTAAAAGAAAAGGGGGGATT
GGGGGGTacagtgcaggggaaagaatagtagaAGATCGGAAGAG
CACACGTCTGAACTCCAGTCAC (8bp custom barcode)
ATCTCGTATGCCGTCTTCTGCTTG-3'

Supplementary Figure 2 | Amplicon sequencing strategy. The sequence of the sgRNA cassette labeled with the binding sites of the sequencing primers.

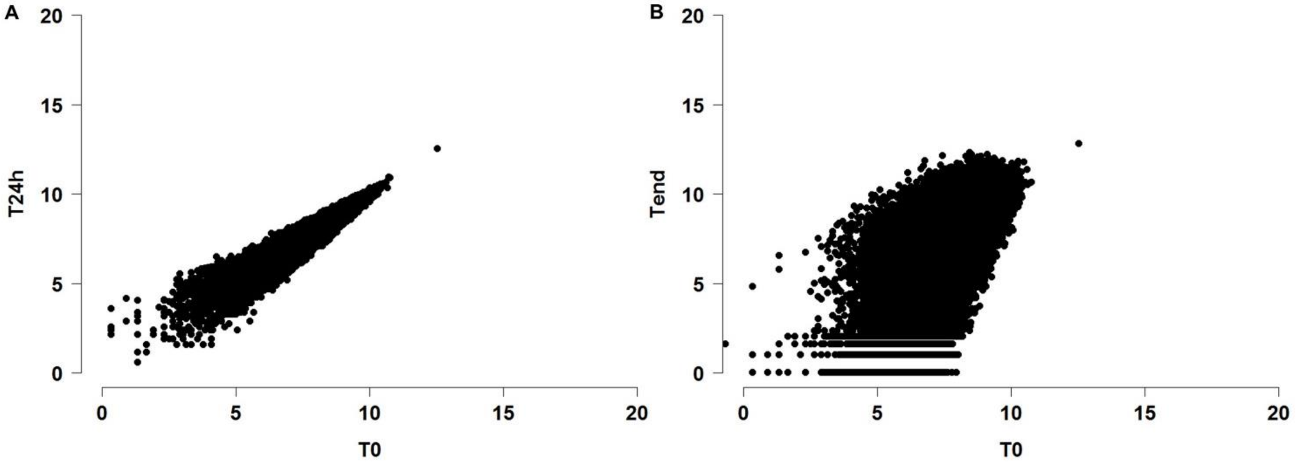

Supplementary Figure 3 | Scatterplots describing the distribution of read counts between samples. a Scatterplot showing enrichment and depletion of Log2 sgRNA read counts after 24h APAP treatment. **b** Scatterplot showing enrichment and depletion of Log2 sgRNA read counts after 4d APAP treatment and 21d outgrowth.

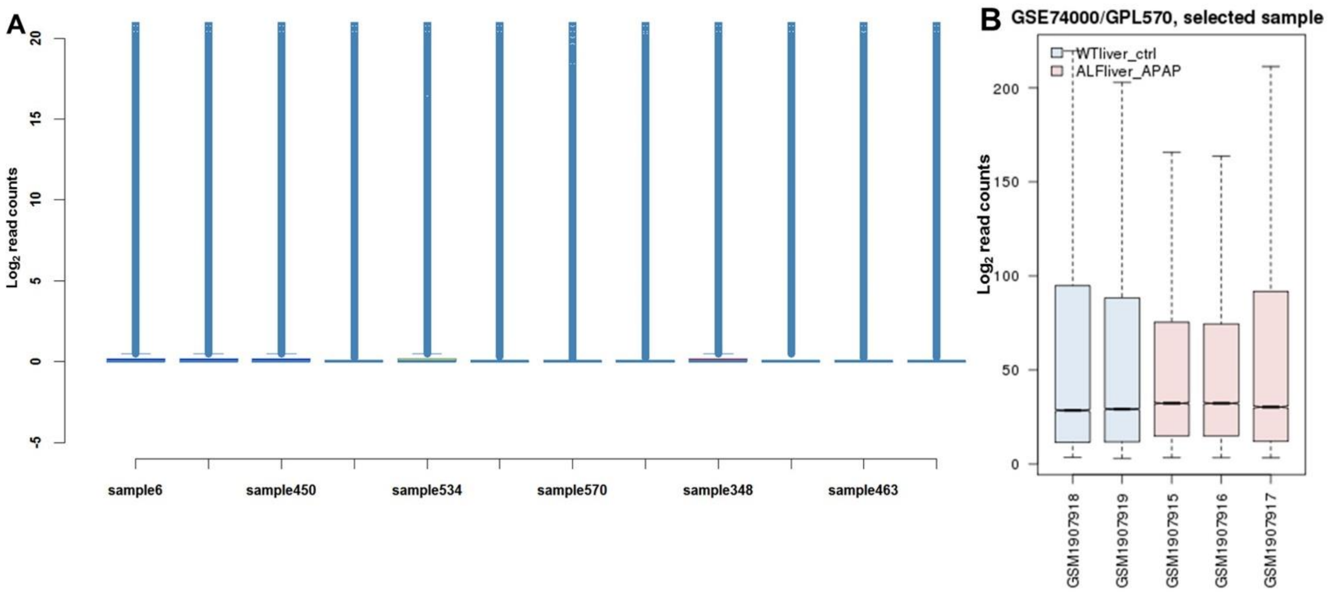

Supplementary Figure 4 | Box plots of mouse RNA-seq (GSE 110787) and human microarray samples (GSE74000) used to validate CRISPR/Cas9 screen hits. a Log_2 read counts of samples with and without APAP treatment from RNA-sequenced mice. **b** Log_2 read counts for GSE74000, healthy liver control and APAP overdose samples.

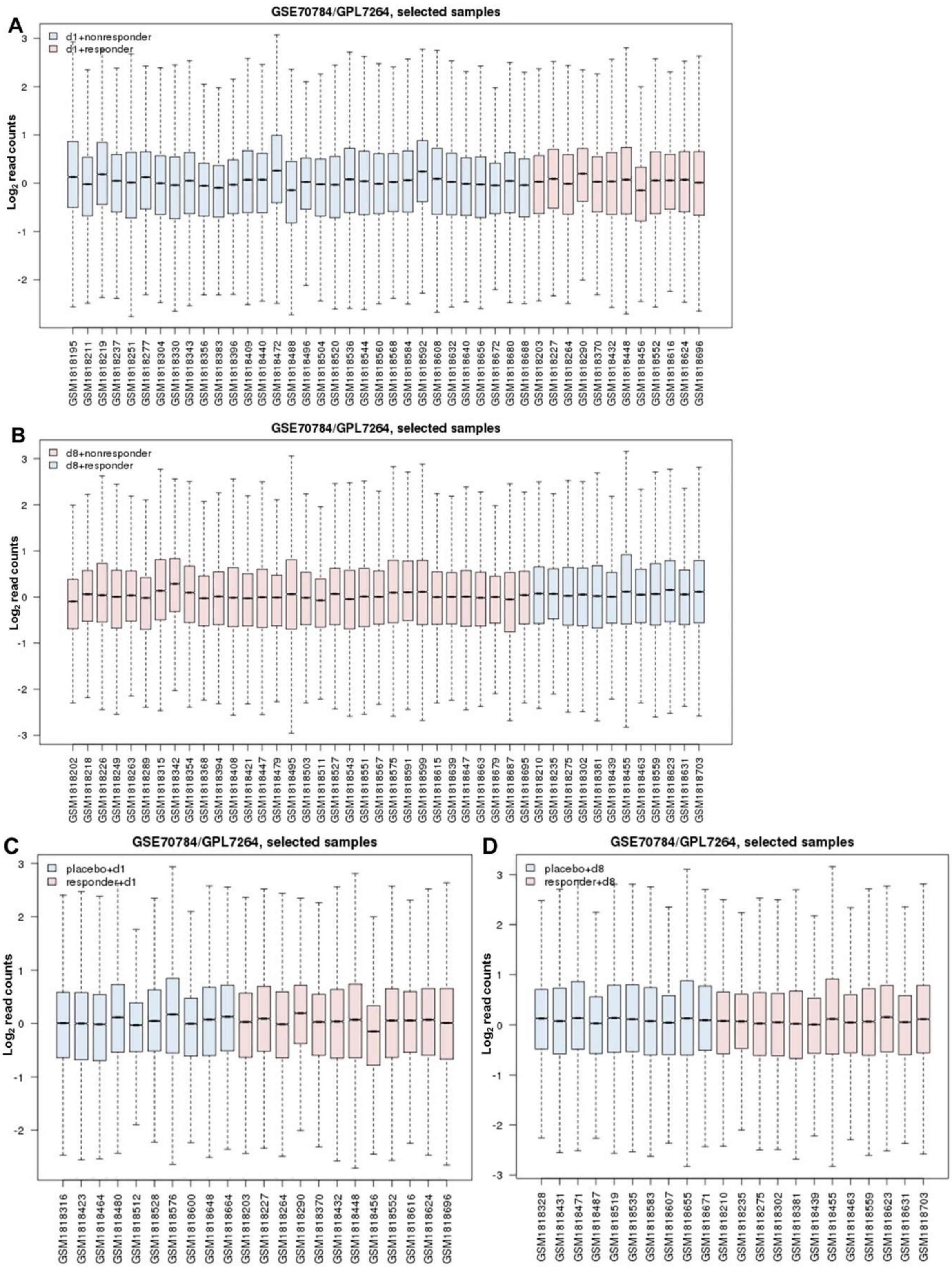

Supplementary Figure 5 | Box plots of human microarray samples (GSE70784) used to validate CRISPR/Cas9 screen hits. a Log_2 read counts from day 1 responder and nonresponder samples in GSE70784. **b** Log_2 read counts from day 8 responder and nonresponder samples in GSE70784. **c** Log_2 read counts from day 1 responder and placebo samples in GSE70784. **d** Log_2 read counts from day 8 responder and placebo samples in GSE70784.

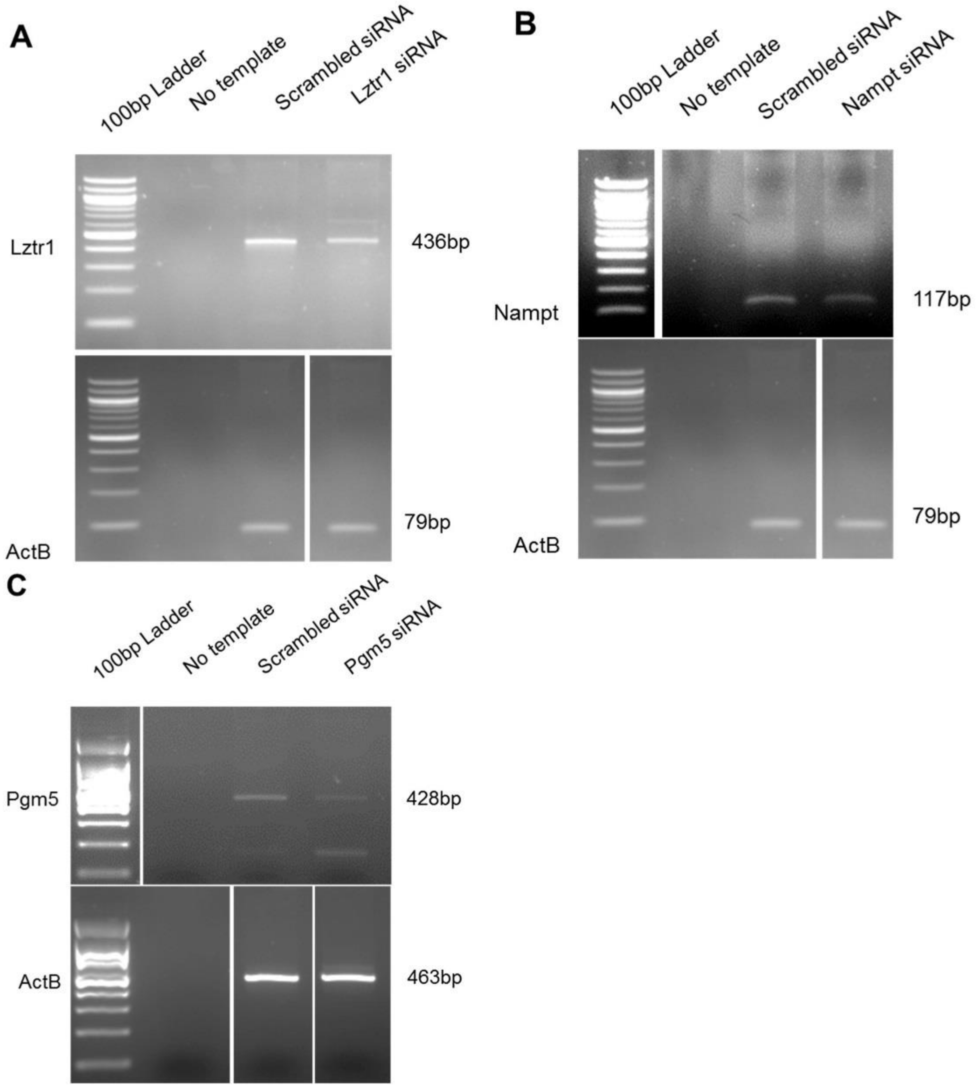

Supplementary Figure 6 | Full-length gels of sqPCR shown in Figure 7. a sqPCR from cDNA prepared from RNA collected 25h post-transfection with 25Mm scrambled or *Lztr1* siRNA. ActB qPCR reactions were conducted concurrently, run on the same gel in non-adjacent lanes, and imaged concurrently. **b** sqPCR from cDNA prepared from RNA collected 25h post-transfection with 25Mm scrambled or *Nampt* siRNA. *ActB* qPCR reactions were conducted concurrently, run on the same gel in non-adjacent lanes, and imaged concurrently. **c** sqPCR from cDNA prepared from RNA collected 25h post-transfection with 50Mm scrambled or *Pgm5* siRNA. *ActB* qPCR reactions were conducted concurrently using the *ActB* F2 and R2 primers, run on the same gel in non-adjacent lanes, and imaged concurrently.

Supplementary Tables

Supplementary Table 1: Alignment metrics for the CRISPR/Cas9 APAP screen.

Label	Total reads /sample	Total mapped reads /sample	Freq mapped genes /sample	Zerocounts /sample
Plasmid_rep1	46103448	35778757	0.78	1
Plasmid_rep2	47102021	37808035	0.80	0
T0_rep1	36989846	20480799	0.55	12
T0_rep2	45321323	36048480	0.80	2
T30min	36441473	24184285	0.66	15
T3h	42968606	34715794	0.81	2
T6h	41412350	33900076	0.82	5
T12h	35096028	27235534	0.78	4
24h_rep1	40517754	14730530	0.36	19
24h_rep2	40473783	32073043	0.79	2
4d_rep1	31545812	10868034	0.34	4957
4d_rep2	42953904	34098492	0.79	1257
Total_rep1	311075317	201893809	0.65	
Total_rep2	175851031	140028050	0.80	
Total	486926348	341921859	0.70	

Supplementary Table 2 Wilcoxon Rank-Sum Test p-values for the CCRISPR/Cas9 screen samples.

Label	Plasmid rep. 1	Plasmid rep. 2	T0 rep. 1	T0 rep. 2	T30min	T3h	T6h	T12h	24h rep. 1	24h rep. 2	4d rep. 1	4d rep. 2
**Plasmid rep. 1**	1.00E+00	9.59E-01	3.83E-03	2.47E-02	5.44E-03	1.96E-01	1.33E-01	1.07E-03	4.16E-01	3.92E-02	7.51E-211	2.63E-66
**Plasmid rep. 2**	9.59E-01	1.00E+00	4.07E-03	2.62E-02	6.13E-03	2.08E-01	1.39E-01	1.06E-03	3.92E-01	4.18E-02	1.77E-211	1.62E-66
**T0 rep. 1**	3.83E-03	4.07E-03	1.00E+00	5.44E-01	8.87E-01	1.34E-01	1.90E-01	5.91E-08	1.37E-03	4.05E-01	3.05E-198	1.86E-56
**T0 rep. 2**	2.47E-02	2.62E-02	5.44E-01	1.00E+00	6.37E-01	3.77E-01	4.69E-01	8.80E-07	8.69E-03	8.41E-01	1.58E-200	4.49E-58
**T30min**	5.44E-03	6.13E-03	8.87E-01	6.37E-01	1.00E+00	1.74E-01	2.31E-01	8.54E-08	2.25E-03	4.83E-01	4.59E-199	5.74E-57
**T3h**	1.96E-01	2.08E-01	1.34E-01	3.77E-01	1.74E-01	1.00E+00	8.52E-01	3.15E-05	6.26E-02	4.81E-01	7.40E-203	3.07E-60
**T6h**	1.33E-01	1.39E-01	1.90E-01	4.69E-01	2.31E-01	8.52E-01	1.00E+00	1.55E-05	4.27E-02	6.03E-01	3.79E-203	1.23E-59
**T12h**	1.07E-03	1.06E-03	5.91E-08	8.80E-07	8.54E-08	3.15E-05	1.55E-05	1.00E+00	2.23E-02	1.38E-06	2.95E-218	5.30E-70
**24h rep. 1**	4.16E-01	3.92E-01	1.37E-03	8.69E-03	2.25E-03	6.26E-02	4.27E-02	2.23E-02	1.00E+00	1.22E-02	3.81E-206	1.74E-62
**24h rep. 2**	3.92E-02	4.18E-02	4.05E-01	8.41E-01	4.83E-01	4.81E-01	6.03E-01	1.38E-06	1.22E-02	1.00E+00	8.10E-203	1.49E-58
**4d rep. 1**	7.51E-211	1.77E-211	3.05E-198	1.58E-200	4.59E-199	7.40E-203	3.79E-203	2.95E-218	3.81E-206	8.10E-203	1.00E+00	3.50E-73
**4d rep. 2**	2.63E-66	1.62E-66	1.86E-56	4.49E-58	5.74E-57	3.07E-60	1.23E-59	5.30E-70	1.74E-62	1.49E-58	3.50E-73	1.00E+00

Supplementary Table 3 | Significant gene hits from the APAP time points (p<0.05) were compared with significantly associated genes from other datasets studying the effects of APAP (GSE74000, ALF healthy liver sample microarray data; GSE70784, d1 and d8 APAP responder and non-responder blood sample microarray data; GSE70784, d1 and d8 APAP responder and placebo blood sample microarray data; and mouse 24h +/- APAP RNA-seq data).

Dataset	4d positive p<0.05	4d negative p<0.05	24h positive p<0.05	24h negative p<0.05	T30min-24h positive p<0.05	T30min-24h negative p<0.05	All positive p<0.05	All negative p<0.05
GSE70784 d1 APAP responder vs. non-responder p<0.05	12	11	15	12	12	19	18	10
GSE70784 d8 APAP responder vs. non-responder p<0.05	98	101	117	94	111	96	100	108
GSE70784 d1 APAP responder vs. placebo p<0.05	22	30	34	20	40	21	31	25
GSE70784 d8 APAP responder vs. placebo p<0.05	91	89	86	68	82	72	72	81
GSE74000 ALFp<0.05	67	63	70	60	81	61	67	57
GSE110787 mouse 24h +/- APAP p<0.05	86	57	63	55	64	58	73	67

Supplementary Table 4 | Top 100 significantly associated genes (p<0.05) from other datasets studying the effects of APAP (GSE74000, ALF healthy liver sample microarray data; GSE70784, d1 and d8 APAP responder and non-responder blood sample microarray data; GSE70784, d1 and d8 APAP responder and placebo blood sample microarray data; and mouse 24h +/- APAP RNA-seq data) were queried in Pubmatrix to determine novelty.

dataset	analysis type	APAP	acetaminophen	hepatotoxic	hepatotoxicity	acute liver injury	acute liver failure
GSE110787 mouse RNA-Seq top 100 genes	Gene expression LFC	15	15	12	24	26	17
GSE74000 top 100 genes	Gene expression LFC	12	12	9	15	15	10
GSE70784 d1 responder vs. nonresponder top 100 genes	Gene expression LFC	8	8	7	14	14	10
GSE70784 d8 responder vs. nonresponder top 100 genes	Gene expression LFC	7	7	4	9	9	7
GSE70784 d1 responder vs. placebo top 100 genes	Gene expression LFC	9	9	6	10	13	8
GSE70784 d8 responder vs. placebo top 100 genes	Gene expression LFC	10	10	14	17	17	13

genes in all top 100 lists	600
unique genes in all top 100 lists	586
unique genes with APAP hits	60

Supplementary Table 5 | Significant gene hits from the APAP time points ($p<0.05$) were compared with a list of 48 genes with known roles in NAD metabolism.

4d pos p<0.05	4d neg p<0.05	24h pos p<0.05	24h neg p<0.05	30min-24h pos p<0.05	30min-24h neg p<0.05	all pos p<0.05	all neg p<0.05
NADK2	*NMNAT1*	*HSD11B1*	*NMNAT1*	*HSD11B1*	*NMNAT1*	*NADK2*	*NMNAT1*
SIRT3		*NADSYN1*		*SIRT1*			
NADSYN1							
SLC36A4							
NUDT9							
SLC25A17							

Supplementary Table 6 | Significant gene hits from the APAP time points (p<0.05) were compared to a list of 133 gene names with known APAP injury-related SNPs.

4d pos. p<0.05	4d neg. p<0.05	24h pos. p<0.05	24h neg. p<0.05	30min-24h pos. p<0.05	30min-24h neg. p<0.05	all pos. p<0.05	all neg. p<0.05
ALCAM	*SUMO1*	*GSS*	*NEB*	*GSTP1*	*CPA6*	*TMPRSS2*	*RAP1GAP2*
GPX4	*KCNJ3*	*IL23R*	*UGT2B15*	*GSS*	*GPX2*	*STAB1*	*SUMO1*
UGT2B15	*SNX16*	*SNX19*	*DNTTIP1*	*STAB1*	*UGT2B15*	*ALCAM*	
	RAP1GAP2	*MAGI2*	*CPA6*	*SLIT2*		*KCNJ3*	
			FBXO39			*MCTP1*	
						TPD52L1	
